# The Prevalence and Regulation of Antisense Transcripts in *Schizosaccharomyces pombe*

**DOI:** 10.1371/journal.pone.0015271

**Published:** 2010-12-20

**Authors:** Ting Ni, Kang Tu, Zhong Wang, Shen Song, Han Wu, Bin Xie, Kristin C. Scott, Shiv I. Grewal, Yuan Gao, Jun Zhu

**Affiliations:** 1 Institute for Genome Sciences and Policy, Duke University Medical Center, Durham, North Carolina, United States of America; 2 Genetics and Development Biology Center, National Heart, Lung and Blood Institute, National Institutes of Health, Bethesda, Maryland, United States of America; 3 DOE Joint Genome Institute, Walnut Creek, California, United States of America; 4 Division of Genomics, Epigenomics and Bioinformatics, Lieber Institute for Brain Development, Johns Hopkins University, Baltimore, Maryland, United States of America; 5 Neuroregeneration and Stem Cell Biology Program, Institute of Cell Engineering, Johns Hopkins University, Baltimore, Maryland, United States of America; 6 Laboratory of Biochemistry and Molecular Biology, National Cancer Institute, National Institutes of Health, Bethesda, Maryland, United States of America; National Institute on Aging, National Institutes of Health, United States of America

## Abstract

A strand-specific transcriptome sequencing strategy, directional ligation sequencing or DeLi-seq, was employed to profile antisense transcriptome of *Schizosaccharomyces pombe*. Under both normal and heat shock conditions, we found that polyadenylated antisense transcripts are broadly expressed while distinct expression patterns were observed for protein-coding and non-coding loci. Dominant antisense expression is enriched in protein-coding genes involved in meiosis or stress response pathways. Detailed analyses further suggest that antisense transcripts are independently regulated with respect to their sense transcripts, and diverse mechanisms might be potentially involved in the biogenesis and degradation of antisense RNAs. Taken together, antisense transcription may have profound impacts on global gene regulation in *S. pombe*.

## Introduction

Emerging evidence has suggested that both strands of eukaryotic genomes are transcribed [1], [2], [3], adding an extra layer to transcriptome complexity. Sense and antisense transcripts are defined as a pair of RNA molecules with significant sequence complementarity [1], [4], [5]. While antisense transcript was first discovered on a gene-by-gene basis [6], bioinformatics analyses of strand-specific ESTs and full length cDNA libraries have provided an glimpse into the genome-wide prevalence of antisense transcripts [1], [7], [8]. Depending on the strategies employed to identify antisense transcripts, their prevalence may vary, ranging from 0.5% to more than 70% [1], [3], [4], [9], [10], [11], [12]. Therefore, reliable methods are required to monitor genome-wide antisense expression and their potential involvement in gene regulation networks.

Several antisense RNAs have been functionally characterized to date, including those involved in X-inactivation, genomic imprinting and clonal gene expression [4], [13]. It has been proposed that antisense transcripts may regulate the expression of their respective sense transcripts through diverse mechanisms [5], [13], [14], such as transcriptional interference, splicing control and mRNA stability. Sense and antisense transcripts might also form a dsRNA duplex to elicit transcriptional and/or post-transcriptional gene silencing [5], [13].

To monitor global antisense expression, several high-throughput strategies have been developed. Pioneering studies relied on microarrays consisting of both strands of the targeted genomic regions [2], [15], [16]. Sequencing-based approach has recently become attractive for comprehensive analysis of transcriptome at unprecedented resolution [17], [18], [19]. Several strand-specific RNA-seq strategies were developed and applied to monitor global antisense expression in different species, including bacterium [20], [21], budding yeast [22], [23], [24], *Arabidopsis* [25], mouse [26], [27], and human cell lines [3], [28], [29]. Since each method has intrinsic limitations that may compromise the overall mapping specificity and efficiency, thus more robust sequencing-based methods are required to reliably monitor antisense transcriptomes.

Herein, we profile antisense transcriptome of *Schizosaccharomyces pombe* with a Directional Ligation sequencing (DeLi-seq) strategy. Initial mapping results showed that nearly one third of protein-coding regions have high-confidence antisense expression, and antisense transcripts are dominate in meiotic and stress-response genes. Detailed analyses further revealed that antisense transcripts are regulated independently of their respective sense transcripts, and multiple distinct mechanisms might be broadly involved in the regulation of antisense expression. Our findings support the notion that gene expression and regulation are more complicated than previously appreciated even in single-cellular organism.

## Results

### DeLi-seq procedure and mapping efficiency

**Figure 1:**
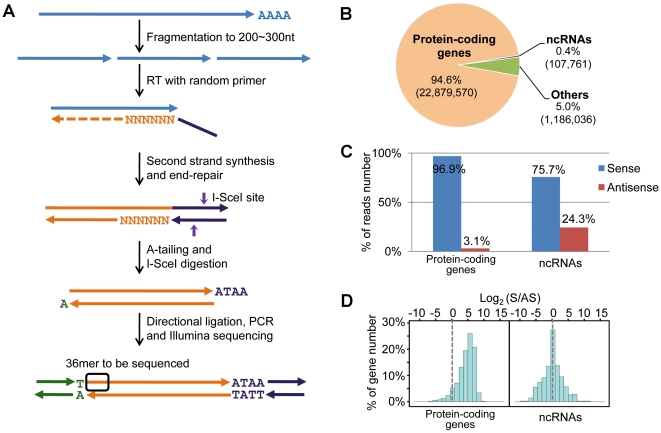
Profiling of antisense transcriptomes by DeLi-seq. (A) Schematic diagram of the DeLi-seq method. PolyA+ RNAs were fragmented by heating with magnesium. Reverse transcription was carried out using random primers with an I-SceI site at the 5′ end. After second-strand synthesis, double-stranded cDNAs were end repaired, followed by adding an “A” base at the 3′ end. The resulting DNA fragments were further digested with I-SceI to generate asymmetric ends, which allow for directional ligation of two different Illumina adaptors. Ligation products were then gel purified to select DNA fragments in 200–300 bp range. Phusion Hot Start High-Fidelity DNA Polymerase was used to produce the final library, which was sequenced using Illumina GAII. (B) Distribution of uniquely mapped reads in different gene categories. (C) The percentage of reads mapped to the antisense orientation for protein-coding genes and ncRNAs. (D) Distribution of the ratio of sense and antisense transcripts for protein-coding genes and ncRNAs.

To monitor sense and antisense transcriptomes simultaneously by high-throughput sequencing, we have developed a directional ligation sequencing strategy or DeLi-seq (Figure 1A). Its overall procedure is similar to that of conventional RNA-seq except that random hexamers required for reverse transcription of polyadenylated RNAs are tagged with a homing enzyme site (I-SceI). After second-strand synthesis, the resulting cDNA fragments are A-tailed followed by I-SceI digestion to generate asymmetric ends. Two Illumina/Solexa adaptors with different overhangs are then directionally ligated so that the strand information of the starting RNA molecules is retained. Thus, DeLi-seq allows for simultaneous detection of sense and antisense transcripts with high confidence.

As a proof-of-concept, we analyzed the transcriptome of *S. pombe* grown under both normal (NM) and heat shock (HS) conditions. We obtained ∼32 million 36-mer raw reads for the four libraries, two biological replicates for each condition. Of them, 96.9% (∼31.3 M reads) were mapped back to the reference genome with 74.8% (∼24.2 M reads) mapped to a unique genomic location (Supplementary information file, Table S1). The rest of the mappable reads are predominantly located in rRNA genes. Of uniquely mapped reads, 94.6% and 0.4% were aligned to known protein-coding genes and non-coding RNAs (ncRNAs), respectively (Figure 1B). As the result, approximately 80% of all annotated genes were covered by >70% of their length (Supplementary information file, Figure S1), indicating that the sequencing coverage is sufficient for monitoring low-abundance transcripts. The remaining unique reads (5.0%) were aligned to genomic locations without previous annotations, suggesting that the annotation of *S. pombe* genome remains incomplete (Figure 1B). 81.6% (3973 out of 4866) of known introns were confirmed by one or more splicing junction reads. We also detected 184 novel introns, and 8 out of 10 selected cases were experimentally validated (Supplementary information file, Figure S2).

### Prevalence of antisense transcripts

We next evaluated the strand specificity of DeLi-seq method with splicing junction reads, which have built-in directionalities. The results showed that 99.4–99.6% of the junction reads are mapped in the correct orientations (Supplementary information file, Table S2), confirming that the DeLi-seq strategy is highly strand-specific. Notably, 3.1% of reads mapped to the antisense strand (∼7–8 fold enrichment over the background level estimated from the junction reads), suggesting that majority of them result from *bona fide* antisense transcripts. Interestingly, the proportion of antisense reads is significantly higher in ncRNAs than in protein-coding genes (Figure 1C and Supplementary information file, Table S3). In fact, ncRNAs tend to have a comparable number of reads on both strands (Figure 1D). Since neither strand of ncRNAs encodes a protein, this observation implies that ncRNAs might function via forming RNA duplexes, which might be subsequently in chromatin remodeling and/or transcriptional gene silencing.

Comparison between biological replicates showed that DeLi-seq results are highly reproducible (*R* = 0.98, Supplementary information file, Figure S3) even for antisense transcripts (*R* = 0.92, Supplementary information file, Figure S4). Negative binomial statistics was then applied (see Materials and Methods for detail) to determine the proportion of protein-coding genes with prominent antisense expression. With a cutoff of *q*<0.01, 2409 genes (or 47.4% of all *S. pombe* protein-coding genes) have antisense expression under normal and/or heat shock conditions (Supplementary information file, Figure S5). Consistent with a recent estimation that 20–49% of human genes have detectable antisense expression [3], our results underscore that antisense expression is a prevalent phenomenon in *S. pombe* genomes.

### Protein-coding genes with dominant antisense expression

**Figure 2:**
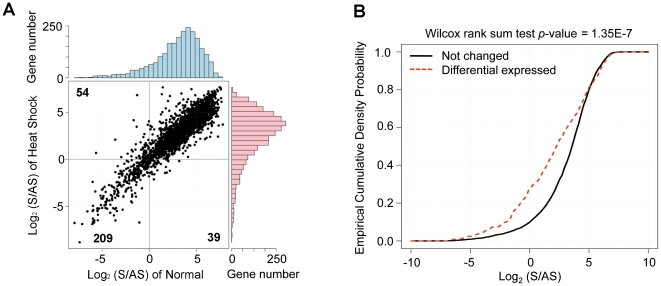
*S. pombe* genes with dominant antisense expression. (A) Dominantly expressed antisense transcripts under NM and HS conditions. Gene number for each quadrant is shown. The distribution of log_2_-transformed sense/antisense ratio of each condition is also shown. (B) Cumulative distribution of the sense/antisense ratio for gene groups, for which the level of sense transcript is either unchanged (black line) or altered (red dotted line) between NM and HS conditions.

The transcribed regions defined by DeLi-seq largely agreed with known genome annotations (Supplementary information file, Figure S6)[30]. However, there are noticeable exceptions of strand orientation. For instance, the majority of the reads at the *SPAC10F6.15* locus were mapped to the *Crick* strand whereas the annotated transcript is on the *Watson* strand (Supplementary information file, Figure S6), indicating that antisense expression might be dominant for at least a subset of genomic regions. In fact, for 302 genes (or 5.9% of all protein-coding genes) the number of antisense reads is equal to or higher than the sense count (Figure 2A). Among them, 209 are detected under both normal and heat shock conditions, and thus are unlikely to result from data irregularity and/or experimental noises. Gene ontology (GO) analysis showed that antisense transcripts are highly enriched in meiotic gene loci (*p* = 5.10E-11) (Supplementary information file, Table S4 and Supplementary information file, Figure S7). Since the yeast strain used in this study is haploid; thus, the expression of meiosis-specific genes is expected to be repressed at transcriptional and/or posttranscriptional level [31]. One possibility is that antisense transcripts may play an essential role in preventing leaky expression of meiotic genes under vegetative condition. Supporting this notion, it has been shown that the expression of *IME4*, an methyltransferase required for initiating meiosis, is repressed by high-level antisense transcripts in haploid budding yeast cells [32].

In addition, dominant antisense expression can also be condition specific. For 54 genes the level of antisense transcripts is higher than that of sense transcripts under the normal but not the heat shock condition. GO analysis showed that these loci are overrepresented in stress response pathways (*p* = 1.20E-13; Supplementary information file, Table S5). One possible explanation is that the expression of sense transcripts (protein-coding) is inhibited by antisense RNAs under normal growth condition. De-repression of antisense-mediated inhibition, possibly in conjunction with transcriptional induction, might allow for quick responses to environmental stress (e.g. heat shock). No functional enrichment was detected for dominant antisense expression specific for heat shock condition, possibly due to fewer genes in this category. In addition, we found the relative abundance of antisense transcripts is considerably higher for differentially expressed genes than those genes whose expression levels remain unchanged (*p* = 1.35E-7, Wilcox rank sum test; Figure 2B). Taken together, these results imply that regulated genes tend to have higher levels of antisense RNAs, which might be involved in the precise control of gene expression.

### Quantitative validation of DeLi-seq results

Stand-specific RNA-seq is expected to facilitate more quantitative transcriptome analysis by avoiding assignment of short sequence reads to the wrong strand (Supplementary information file, Text S1 and Figure S8). To evaluate how quantitative is the expression level determined based on DeLi-seq read count, we compared our results with the strand-specific transcriptome data generated by HybMap [16]. For sense transcripts, the two data sets largely agreed with each other (*R* = 0.63; Supplementary information file, Figure S9A). This is comparable to a recent report where RNA-seq and tilling array were performed side-by-side (*R* = 0.68)[30]. In contrast, DeLi-seq clearly detected more antisense transcripts than the HybMap approach (Supplementary information file, Figure S9B), suggesting that the sequencing-based approach is more sensitive for detecting low-abundance transcripts than hybridization-based platforms.

**Figure 3:**
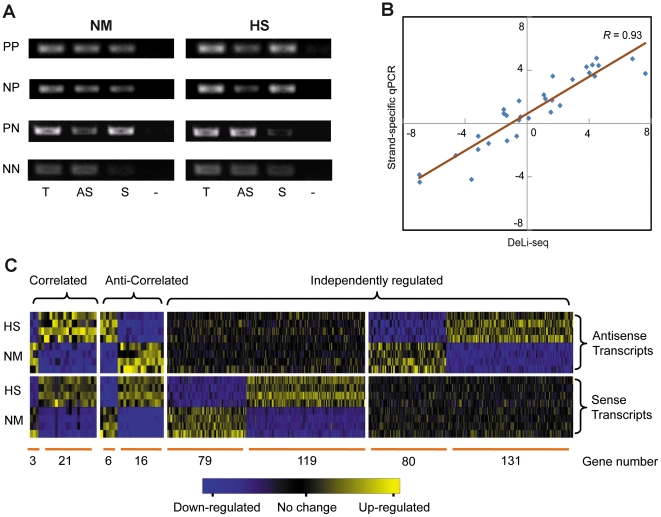
Differentially expressed sense and antisense transcripts. (A) Strand-specific RT-PCR results of four loci selected for validation. PCR products specific to antisense (AS) or sense (S) transcripts are shown. Positive control (T; RT with two gene-specific primers) and negative control (-; no primer at RT step) are also included. PP: S≥ AS in both normal (NM) and heat shock (HS) conditions; PN: S≥ AS in NM and S< AS in HS; NP: S< AS in NM and S≥ AS in HS; NN: S< AS in both NM and HS. (B) Correlation between DeLi-seq and strand-specific qPCR. The log_2_(S/AS) values obtained by DeLi-seq (X axis) and the ΔCt values between sense and antisense transcripts determined by quantitative strand-specific RT-PCR (Y axis) were used to compute the correlation coefficient (*R*). (C) Sense and antisense transcripts are either coordinated (correlated or anti-correlated) or independently regulated. The number of genes in each category is shown.

Strand-specific RT-PCR was also employed to evaluate DeLi-seq results. We randomly selected sixteen genes for which the sense/antisense ratios are broadly distributed among the NM and HS conditions. Amplification products were first resolved by gel electrophoresis, which confirmed the existence of antisense transcripts as well as assay specificity (Figure 3A and Supplementary information file, Figure S10). Strand-specific qPCR was then performed, which show highly correlated results with DeLi-seq (*R* = 0.93, Figure 3B and Supplementary information file, Table S6). Together, these results demonstrated that DeLi-seq is a reliable method for quantitative analysis of sense and antisense transcriptomes.

### Sense and antisense transcripts are independently regulated

We next aimed to identify differentially expressed sense and antisense transcripts by focusing on 2409 loci with antisense expression. This resulted in 257 antisense transcripts whose expression levels are significantly altered between the two conditions (>2-fold change, *q*<0.05, Figure 3C). Although coordinated expressions were detected for a small subset of the loci, majority of the sense and antisense transcripts tend to be independently regulated (Figure 3C). Further analyses revealed that the correlated and anti-correlated groups show similar level of relative antisense expression as those independently regulated groups, suggesting that the observation is not due to differential antisense levels among these groups (Supplementary information file, Figure S11). Our result is in contrast to a previous report[16], which suggested a positive correlation between sense and antisense transcripts in fission yeast. One major difference is that our study focused on polyadenylated transcripts while the earlier study did not distinguish transcripts with or without polyA tail. Therefore, it is suggestive that polyadenylated antisense transcripts may behave/function differently compared to those without a polyA tail. More importantly, independent regulation of sense and antisense expression implies that antisense-mediated gene regulation might (1) occur at the posttranscriptional levels (e.g., mRNA translation and/or localization) without affecting the abundance of sense transcripts; or (2) function *in trans* to regulate gene at distant location.

### Correlation between antisense transcription and Pol II occupancy

**Figure 4:**
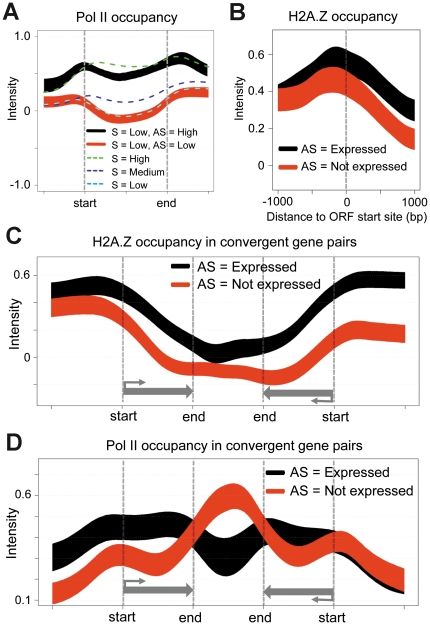
Antisense transcripts have distinct Pol II and H2A.Z occupancy. (A) Pol II occupancy in protein-coding region (ORF), and 1 kb regions upstream and downstream of ORF. The start and the end positions of ORF (sense strand) are indicated. The gene groups defined by sense expression (high, medium and low) are drawn as dashed lines. The weakly expressed genes were further divided into antisense- (black) and sense-dominant (red) groups based on sense/antisense ratio. (B) H2A.Z occupancy at the ORF start site and its surrounding regions. Two gene groups, i.e. genes with antisense expression (black) and without antisense expression (red) are shown. (C) H2A.Z and (D) Pol II occupancy for convergent loci. Two gene groups, i.e. genes with antisense expression (black) and without antisense expression (red) are shown. Compilation of two gene groups (comparable expression distribution of sense transcripts; and presence/absence of antisense expression) were based on the left genes in the convergent pairs. For completeness, the right genes are also shown, which do.

One interesting question is whether antisense transcripts, similar to sense transcripts, are generated by RNA Polymerase II (Pol II). We thus compared the DeLi-seq results with Pol II occupancy [33] to further corroborate antisense transcription. Pol II occupancy in general correlates well with the expression level of sense transcripts (Figure 4A). This is expected because sense transcripts are dominant for most *S. pombe* genes and would overwhelm the contribution of antisense transcripts. We then focused on weakly expressed sense loci (bottom third of all genes), for which potential antisense transcription would be more prominently reflected by Pol II. These loci were further divided into two groups based on the relative ratio of antisense/sense transcripts (AS/S). The results showed that Pol II occupancy is significantly higher in loci with high-level antisense transcripts than those with lower AS/S ratio (Wilcoxon rank test *p*<1E-200; Figure 4A). Thus, our finding strongly suggests that polyadenylated antisense transcripts are the result of active transcription.

### Antisense expression is mediated by bidirectional promoter and transcriptional read-through

Independent regulation of sense and antisense transcripts implies that antisense transcripts are autonomously expressed, either with their own promoters or through other mechanisms. Emerging evidence suggests that bidirectional promoters are likely a major contributor for antisense expression in budding yeast [34], [35]. Under this scenario, two sense genes are orientated in tandem, and a bidirectional promoter simultaneously drives the expression of the upstream antisense transcripts and the downstream sense gene. We found that such a mechanism also exists in *S. pombe* as exemplified at the *Guf1*/*Wis2* locus (Supplementary information file, Figure S12). Genome-wide analysis of tandem gene pairs showed that the level of antisense RNAs does not correlate with their own sense transcripts but rather with the downstream sense genes (*p*<5.36E-07; Pearson correlation), suggesting that bidirectional promoters might be involved in antisense transcription in at least a subset of tandem gene loci.

Transcriptional read-through is another mechanism underlying antisense expression [33], [36]. In this case, the two sense genes are organized in a convergent or “tail-to-tail” orientation (Supplementary information file, Figure S13A). The read-through transcripts of one gene would potentially become the antisense transcripts of the other gene transcribed from the opposite direction. One example is the *S. pombe* gene pair encoding the GRIP- and LEA domain-containing proteins, respectively. Using strand-specific RT-PCR, we detected the read-through transcript originated from the LEA gene (Supplementary information file, Figure S13B). Global analysis of convergent loci showed that the abundance of read-through transcripts (or antisense RNAs) is significantly correlated with the level of their upstream transcripts (*p*<6.15E-09; Pearson correlation). Taken together, these results suggest that transcriptional read-through, similar to bidirectional promoter, might partially explain antisense expression at the convergent loci in *S. pombe*.

### H2A.Z and antisense expression at convergent loci

H2A.Z, a widely-conserved histone variant, is often found in the nucleosome free regions (NFRs) of eukaryotic promoters and involved in transcriptional gene regulation[33], [37]. Interestingly, it has recently been shown that H2A.Z might also play a role in suppressing read-through antisense transcripts in fission yeast[33]. H2A.Z occupancy was therefore analyzed for genes with or without antisense expression. To avoid potential complications due to differential gene expression levels, the two gene groups were compiled in such a way that they have the same number of genes and nearly identical expression distributions of sense transcripts. We found that the H2A.Z level is considerably higher in genes with antisense expression than those without (Wilcoxon rank test *p*<1E-22; Figure 4B). Similar analyses were also performed for convergent, divergent and tandem loci. The phenomenon is more prominent in convergent and tandem loci compared to divergent gene pairs (Figure 4C and data not shown). Supporting the previous report[33], our results imply that H2A.Z might be a general indexing factor that marks antisense expression. Because the major difference in H2A.Z occupancy was observed in the gene body than the promoter region, it indicates that H2A.Z modulates antisense expression at a step other than transcriptional initiation (e.g. degradation).

Pol II occupancy was then examined with respect to the presence of antisense expression. We focused on convergent loci because antisense transcript, if there is any, is presumably the result of transcriptional read-through rather than driven by its own promoter. Therefore, the difference in Pol II occupancy (the above H2A.Z occupancy as well) can be interpreted without the complication of extra promoter activity. Strikingly, we found that the Pol II occupancy tends to be higher in the transcribed region of genes with antisense expression than those without (Figure 4D), similar to what was observed for H2A.Z occupancy. In contrast, Pol II is accumulated at the intergenic region between convergent gene pairs, and this phenomenon was only observed in the gene group without antisense expression. Elevated Pol II level in the intergenic region is likely the result of transcriptional termination. Improper termination will lead to transcriptional read-through and ultimately, antisense transcripts. This may explain why convergent gene pairs with antisense expression exhibit reduced Pol II occupancy at the intergenic regions.

Two possibilities might be plausible for increased Pol II occupancy at transcribed regions: (1) elongating Pol II associated with antisense transcription; or (2) stalling of Pol II due to antisense expression. We favor the second model in that the relative contribution of antisense transcription (compared to sense transcription) to Pol II occupancy is expected to be small. More importantly, H2A.Z occupancy is also higher at these antisense-containing regions, suggesting co-existence of H2A.Z and stalled Pol II (Figure 4C, 4D). Although it is unclear whether H2A.Z is causal or the consequence of Pol II stalling, one attractive model is that H2A.Z may directly or indirectly signal the Pol II-associated exosome to degrade antisense transcripts (33, 38). Furthermore, this mechanism might be also involved in regulating promoter associated short transcripts (see **Discussion**), thereby severing as a general quality control mechanism for the *S. pombe* transcriptome.

## Discussion

Assisted by strand-specific RNA-seq technology, we provide evidence that antisense expression is prevalent in the *S. pombe* transcriptome. For 302 (or 5.9%) protein-coding genes, the abundance of antisense transcripts is higher than their respective sense transcripts. Because antisense expression is condition-specific (Figure 2A), one would expect more such instances if additional conditions are analyzed. We further showed that differentially expressed sense and antisense transcripts tend to be independently regulated, and that bidirectional promoter and/or transcriptional read-through are two common mechanisms that drive antisense expression. Unique for strand-specific RNA sequencing approaches, DeLi-seq improves the profiling of sense genes by avoiding erroneous assignments of antisense reads. Therefore, whether antisense transcript is of interest or not, strand-specific transcriptome sequencing approaches are better suited for quantitative analysis of transcriptome profiles than conventional RNA-seq methods.

The sequencing data employed in this study consists of 36-mer reads derived from a defined end of the target cDNA molecules. The data acquisition step can be improved by incorporating the latest developments in sequencing technology to obtain paired-end and/or longer sequence reads. Such improvements are expected to better reveal the complexity of eukaryotic transcriptomes, such as alternative splicing variants and other posttranslational regulatory events.

Independent regulation of sense and antisense transcripts implies that antisense transcripts may have their own promoters or transcribed by other means. We provide initial evidences that bidirectional promoter and transcriptional read-through might in part explain antisense expression in genomic loci with tandem and convergent orientation, respectively. Other mechanisms may also exist, such as antisense-specific promoters that are independent of nearby sense genes. We speculate that antisense transcripts constitute a heterogeneous group of regulatory ncRNAs. Further investigations are warranted to characterize the biogenesis as well as the regulation of this hidden layer of transcriptome.

We provide evidence that H2A.Z occupancy is significantly increased in gene loci with antisense expression, supporting the notion that H2A.Z might be an indexing factor that mediates suppression of antisense transcripts. It has been shown exosome components may directly or indirectly interact with H2A.Z (33) and elongating Pol II (38). Our results, which showed elevated H2A.Z level tends to coincide with increased Pol II occupancy in the presence of antisense transcripts, suggest that H2A.Z might signal Pol II-associated exosome to degrade antisense transcripts at the convergent loci. In addition, a number of studies showed that promoter-associated short RNA (PASR) is a widespread phenomenon of active loci[38], [39], [40]. Because PASRs are often bidirectionally transcribed, the high level of H2A.Z observed at the promoter-proximal regions may also tag the PASRs for degradation. In addition, H2A.Z is also involved in transcriptional regulation by forming unstable nucleosome at the promoter regions[41]. We speculate that these two functions are not necessarily mutually exclusive. Instead, H2A.Z might play a dual role to coordinate transcriptional activation and RNA surveillance. Further investigations are required to characterize the functions of H2A.Z and antisense transcripts in transcriptional regulation.

Lastly, this study is focused on long polyadenylated antisense transcripts. Our results showed that they might play a role in gene regulation distinct from those antisense RNAs without polyA tails [16]. In fact, antisense transcriptomes are expected to be more complex, consisting of RNA molecules of different sequence/structure characteristics (e.g. capped or non-capped), cellular locations (e.g. nuclear vs. cytoplasmic), lengths (long or short) and stabilities [1], [42], [43], [44], [45], [46]. Future efforts are required to systematically identify and characterize antisense transcripts of different classes. Using methods like DeLi-seq to generate a comprehensive inventory of transcriptome will facilitate a better understanding of eukaryotic gene regulation.

## Materials and Methods

### DeLi-seq library construction

Fission yeast was cultured in rich medium with or without heat shock (see Supplementary information file, Methods S1 for detail). Total RNA was isolated by a hot phenol procedure[47]. Polyadenylated RNA was then prepared with two rounds of polyA selection using Dynabeads Oligo (dT)25 (Invitrogen) according to a modified protocol (Supplementary information file, Methods S1). 1 µg of PolyA+ RNA was dissolved in 30 µl fragmentation buffer (40 mM Tris-HAc (pH 8.2), 100 mM KAc and 30 mM MgAc_2_) and heated at 94°C for 3 min. RNA fragments were precipitated with GlycoBlue (Ambion) as a carrier. Reverse transcription (RT) of the recovered RNA was performed with SuperScript II reverse transcriptase (Invitrogen) in a 50 µl reaction, containing 2.5 pmol random primer (5′-AGA CAT TAC CCT GTT ATC CCT ANN NNN N-3′), 0.25 pmol oligo(dT) primer (5′-AGA CAT TAC CCT GTT ATC CCT ATT TTT TTT-3′), 100 units of RNasin (Promega) and 6 ng/µl freshly-made actinomycin D (which inhibits DNA-dependent DNA polymerase activity of reverse transcriptase[48]). RT reaction was incubated at 25°C for 10 min, 42°C for 60 min and 75°C for 15 min. First-stand cDNAs were then purified by ZYMO DNA clean & concentrator-5 kit. After second-strand synthesis, double-stranded cDNAs were end repaired by T4 DNA polymerase, followed by A-tailing with Klenow DNA polymerase (exo-). The resulting DNA fragments were further digested with I-SceI to generate asymmetric ends, which allow for directional ligation of two different Illumina linkers (Supplementary information file, **Methods S1**). Ligation products were then gel purified to select the DNA fragments in the 200–300 bp range. 12-cycle PCR was then performed with Phusion Hot Start High-Fidelity DNA Polymerase (Finnzymes) to generate the final sequencing library, which was sequenced using Illumina/Solexa Genome Analyzer II.

### Strand-specific RT-PCR

Total RNA was isolated from *S. pombe* cells by a hot phenol procedure. RNeasy Mini kit (QIAGEN) was used to remove potential genomic DNA contamination; and a DNase I digestion step was also included. 250 ng of DNA-free RNA was used to perform reverse transcription (RT) with gene-specific primers. Since antisense and sense transcripts are unlikely to share same introns, we only use primer pairs span single exon to perform the validation. 30 cycles of PCR was performed in 20 µl reaction, which contains 1 µl of RT reaction, 1x AmpliTaq Gold buffer (ABI), 1.5 mM MgCl2, 0.2 mM dNTP, 3 pmol of forward primer, 3 pmol of reverse primer and 1.5 unit AmpliTaq DNA polymerase. We preferred to use AmpliTaq Gold DNA polymerase (ABI) to perform qPCR because the hot start performance of the enzyme provides higher specificity of amplicons. To monitor the amplification curve, 0.15 µl 10x SYBR Green I (Molecular Probe) was added to the 15 µl PCR reaction described in strand-specific RT-PCR. Each sample was prepared in duplicate to get more reliable Ct value.

### Raw data mapping

The genomic sequence (Sep. 2008 version) and genome annotation (gff file, July. 2008 version) of *S. pombe* was downloaded from GeneDB (ftp://ftp.sanger.ac.uk/pub/yeast/pombe/GFF/). A three-step procedure was then used to map raw sequence data to the reference pombe genome. Step 1: Short tags were mapped to the genome sequence using SOAP program[49] and a maximum of 2 mismatches was allowed; Step 2: Short reads that cannot be mapped in step 1 were mapped to all possible junctions (which are generated based on all annotated exons in *S. pombe*) using SOAP; Step 3, Unmapped reads in steps 1 and 2 were mapped to the *S. pombe* genome using BLAT with the parameters of ≥95% identity and gap size ≤2000 bp (potential introns). Only uniquely mapped reads were used in the following analysis, and the definition of sense and antisense transcripts are based on known genome annotation. For 43 coding-coding pairs that are annotated to have partial 5′-5′ or 3′-3′ overlaps, approximately 0.04% of the all reads mapped to these regions. These ambiguous reads were eliminated to avoid double counting. To visualize the reads along the fission yeast genome, wiggle files were generated from uniquely mapped reads based on their location and counts.

### Graphic view of mapped reads

The coverage of each individual base in the genome was determined by normalizing to the total number of uniquely mapped reads for each condition and the results were stored in wiggle format. The data were then visualized by two methods: (a) we constructed a local gff2 database that integrates the reference genome sequence, known annotations and wiggle files generated from our dataset. The results can be visualized by GBrowse (GMOD project) and a local gff2 database; (b) Using a modified plotAlongChrom function in the BioConductor “tilingarray” package, base counts were plotted along genome annotation in a given region. A 16-bp window was used to perform data smoothing. Figure S6 and S7 were drawn based on the second strategy.

### Gene expression analysis

The genome annotations of *S. pombe* were downloaded from GeneDB (version 7/16/08). For each given protein-coding gene or noncoding RNA, the normalized count of reads uniquely mapped to the region was first computed. To determine the relative expression level, the read count was further normalized by the transcript length and mappability (189,359 of bases would not be covered due to low sequence complexity). Overall, 5079 protein-coding genes and 491 previously reported ncRNAs[30] were included in the analysis. To compute the expression level of antisense transcript, the same strategy was used except that the intronic region, if there is any, in the antisense direction of a given protein-coding gene was considered as expressed region. This is because that antisense transcript is expected to have a different intron/exon structure as its sense transcript, and we did not find significant number of introns in the antisense fragments.

### Differential gene expression analysis

Gene expression values were normalized based on the total number of uniquely mapped reads of each library. In order to increase the overall accuracy, the results of four additional libraries were included, which are technical replicates of the 4 libraries shown in the main text. These four libraries have fewer reads (due to cluster generation step, data not shown); however, their gene expression profiles are well correlated with their respective technical replicates. To define differentially expressed transcripts between wide type and heat shock conditions, SAM (significant analysis of microarray, siggenes package from BioConductor) was employed to compute the *q* value. A stringent criterion (*q*<0.05 and fold changes >2) was used to identify transcripts whose expression levels were significantly changed.

### Gene ontology (GO) analysis

GO enrichment analysis was performed with the topGO package (BioConductor), and GO annotations for *S. pombe* genes was download from GeneDB[50]. One-sided Fisher's exact test was used to determine the *p* value for the enrichment of a given functional category.

### Analyses of Pol II and H2A.Z occupancy

ChIP-chip datasets of Pol II and H2A.Z were downloaded from GEO with accession ID GSE17271. Both Pol II and H2A.Z intensity was normalized by whole-cell genomic DNA. The localization of the probe was lifted to match the updated *S. pombe* genome (Sep. 2008 version) in GeneDB. Genes with CDS less than 200 bp were excluded from the analyses. Probes fell in gene body, 1000 bp upstream of ORF start site and 1000 bp downstream of ORF end site were used to analyze the pattern of Pol II and H2A.Z. Gaussian filter (sd  = 40 bp) was used to smooth the data points. In the generated graphs, the height and the thickness of each curve represents the mean value and standard deviation of the local data points.

#### Additional methods

Detailed DeLi-seq library construction protocols and computational analyses are available in the Supplementary information file, Methods S1. Raw data were deposited at the NCBI Short Read Archive with accession # SRA026539.1.

## Supporting Information

Text S1 **Strand information facilitates more quantitative transcriptome analysis.**
(DOC)

Methods S1 **Supplementary Methods.**
(DOC)

Figure S1 **Overall coverage of sense transcripts.**
For each annotated protein-coding gene in *S. pombe*, the coverage at the nucleotide level was computed based on the DeLi-seq reads uniquely mapped to the locus in the sense orientation. Cumulative plot is used to show the percentage of genes that passes each respective coverage threshold. Four DeLi-seq libraries were analyzed separately and the results showed that they have comparable coverage depth.
(TIF)

Figure S2 **Validation of novel introns.**
10 candidate novel introns were selected for validation. For each candidate, a pair of gene-specific primers was designed upstream and downstream of the putative intron. RT-PCR was performed with the RNA samples obtained from normal or heat shock condition. Genomic DNA was used as a negative control, which gives rise to unspliced products. For 8 out the 10 cases, spliced products with an expected size were observed. In the case of *SPBP35G2.04c*, two novel introns were identified by DeLi-seq, and one of them was randomly selected for validation.
(TIF)

Figure S3 **Reproducibility of the DeLi-seq method.**
The count of uniquely mapped reads for each annotated locus was determined and normalized to the total number of reads of each library. Sense and antisense transcripts of each locus were treated as separated data points. Correlation coefficient was then computed between the two biological replicates of either normal (A) or heat shock (B) condition.
(TIF)

Figure S4 **Reproducibility of sense or antisense read counts.**
Pairwise comparison of the normalized expression level of sense (A) or antisense (B) transcripts among four DeLi-seq libraries. The histograms on the main diagonal represent the expression distribution of individual libraries. Each scatter plot was generated based on the normalized gene expression levels obtained from the two corresponding libraries. Pearson correlation coefficients are shown for all possible library pairs.
(TIF)

Figure S5 **Identification of gene loci with high-confidence antisense expression.**
Since the overall log_2_(S/AS) follows a negative binomial distribution (Figure 2A), we thus used negative binomial statistics to remove low-confidence call of antisense transcripts. The background rate of each library was experimentally defined based on the splicing junction reads. Five different thresholds were used as indicated.
(TIF)

Figure S6 **Visualization of DeLi-seq results.**
A 30 kb genomic region is shown with known annotations (upper panel), including protein-coding genes (light blue) and ncRNAs (green). In the lower panel, read counts from the top strand (orange) and bottom strand (dark blue) are shown separately. For SPAC10F6.15 locus, the level of antisense transcripts is much higher than that of sense transcripts (open box).
(TIF)

Figure S7 **Dominant antisense expression at the *spo6* locus.**
*Spo6* gene is encoded on the Watson strand based on genome annotation. However, majority of the reads were mapped to the Crick strand in this locus under both normal and heat shock conditions.
(TIF)

Figure S8 **Comparison between DeLi-seq and HybMap results.**
For DeLi-seq method, the relative expression level of each transcript was computed based on normalized read count. HybMap data was downloaded from http://bioserver.hci.utah.edu/SupplementalPaperInfo/2008/Dutrow_NatGen_PombeTranscriptome/. The data set contains the expression values that were computed based on the probe intensity subtracted against the background (intergenic regions). Pearson correlation coefficient (*R*) was computed for both sense (A) and antisense (B) transcripts.
(TIF)

Figure S9 **Validation of antisense expression by strand-specific RT-PCR.**
16 genes were randomly selected to examine antisense expression. These genes are broadly divided into four different categories based on the ratio of sense (S) and antisense (AS) transcripts (**PP**: S≥ AS in both NM and HS; **PN**: S≥ AS in NM and S< AS in HS; **NP**: S< AS in NM and S≥ AS in HS; **NN**: S< AS in both NM and HS). To carry out strand-specific RT-PCR, either the forward or reverse primer was added at the RT step, which specifically amplifies the antisense or sense transcripts, respectively. As a positive control, both primers were added at the RT step (T). In addition, RT reaction was also performed without any primer to serve as a negative control (−). The final PCR products were resolved by agarose gel electrophoresis, and each of the primer pairs gave rise to a specific band with expected size.
(TIF)

Figure S10 **Comparison of antisense expression level in differentially expressed genes.**
Differentially expressed genes were divided into two groups based on sense-antisense expression correlation. The correlated group (Cor) contains sense-antisense pairs with correlated or anti-correlated expression patterns. The non-correlated group (Non-Cor) group consists gene loci for which sense and antisense transcripts are independently regulated. The absolute antisense expression level (A) or relative antisense/sense ratio (B) was then compared between the two groups by one-way ANOVA test. No significant difference can be detected between the two subcategories in terms of antisense expression level (p = 0.125) or antisense/sense ratio (p = 0.776).
(TIF)

Figure S11 **Bidirectional promoter leads to antisense transcription in tandem gene pair.**
(A) A Schematic diagram of the *Wis2-Guf1* locus. The sense transcripts of these two genes (solid boxes) are encoded on the negative strand of chromosome 1, and organized in a tandem orientation. Based on the DeLi-seq results, the expression of *Guf1* antisense transcripts (dashed box) are increased under the heat shock condition and the increase is coordinated with *Wis2* sense transcript. *Wis2* encodes a peptidyl-prolyl isomerase required for protein unfolding, transport and assembly. The *Guf1* sense transcript encodes a mitochondrial matrix GTPase associated with mitochondrial ribosome, and is known to be down-regulated by heat shock. (B) Validation of DeLi-seq results for the *Wis2-Guf1* locus by strand-specific RT-PCR. T, combined level of sense and antisense transcripts; A: antisense transcripts; S: sense transcripts; -, negative control, for which no primer was added during reverse transcription. (C) A conventional heat shock element (HSE) with multiple GAA blocks is identified in the candidate bidirectional promoter region. Both sense transcript of *Wis2* and antisense transcript of *Guf1* have their own TATA-box (black block).
(TIF)

Figure S12 **Antisense transcripts derived from transcriptional read-though at a convergent locus.**
(A) Schematic diagram of the *SPBC365.11* and *SPBC365.12c* loci. The two sense transcripts (solid box) are coded on different strands and organized in a convergent orientation (tail-to-tail). The expression level of both *SPBC365.12c* and the antisense transcripts to *SPBC265.11* (dashed box) are induced by heat shock. In contrast, the sense transcript of *SPBC365.11* is transiently down-regulated under the heat shock condition. (B) Heat shock induced transcriptional readthrough is confirmed by strand-specific RT-PCR. T, Total level of sense and antisense transcripts; AS, expression level of antisense transcripts; S, expression level of sense transcripts; -, negative control, which contained no primer during reverse transcription. In order to detect readthrough transcripts, a primer pair was used which spans the two annotated genes; the relative locations of these two primers are shown. At the RT step, only left (L) or right (R) primer was added to detect readthrough transcript of right (*SPBC365.12c*) or left (*SPBC365.11*) gene. The readthrough transcript of *SPBC365.12c* gene was apparent under the heat shock condition.
(TIF)

Figure S13 **Differentially expressed sense and antisense transcripts identified by DeLi-seq.**
(A) Differentially expressed sense transcripts (*q*<0.05 and >2-fold change) are shown as colored dots in a MA plot. Up-regulated (red and blue) and down-regulated (green and magenta) are shown. False negative (blue and magenta) and false positive (black) genes were determined assuming strand information is not provided. (B) The number of false positives and false negatives obtained in (A) are color coded and shown in a Venn diagram.
(TIF)

Table S1 **Mapping efficiency of sequencing reads.**
(DOC)

Table S2 **The reads mapped to the exon-exon junctions in sense and antisense transcripts.**
(DOC)

Table S3 **Uniquely mapped reads in protein-coding region and ncRNAs.**
(DOC)

Table S4 **GO analysis of genes with AS ≥S in both NM and HS.**
(DOC)

Table S5 **GO analysis of genes with AS ≥S in NM but not HS condition.**
(DOC)

Table S6 **Primers used for strand-specific RT-PCR and qPCR.**
(DOC)
