# Supplementary material for: The Prevalence and Regulation of Antisense Transcripts in Schizosaccharomyces pombe
Source: PLoS One. 2010 Dec 20;5(12):e15271. doi: 10.1371/journal.pone.0015271 (PMC3004915; doi:10.1371/journal.pone.0015271)
Supplement: Text S1 — Strand information facilitates more quantitative transcriptome analysis. (DOC) [file pone.0015271.s001.doc]

**Supplementary information file: Text S1**

**Strand information facilitates more quantitative transcriptome analysis**

Assured by the validation results, we aimed to identify differentially expressed sense transcripts. Exposing cells to heat caused significant changes in expression of a large number of coding genes (> 2-fold change and *q* < 0.05). As expected, the upregulated genes (301 total) are overrepresented in cellular processes involved in stress response (185/301, *p* < 1E-30). The downregulated genes (181 total) are enriched in rRNA processing (48/181, *p* < 1E-27) and ribosome biogenesis (75/181, *p* < 2.1E-18), consistent with the notion that active protein synthesis is blocked under the heat shock condition.

It is worth noting that the above analysis was performed with prior knowledge of sense and antisense strands. Assuming that strand information is not provided, antisense reads would be mistakenly assigned to the sense strand (e.g. conventional RNA-seq). This will introduce noise to expression analysis, especially for those genes with high-level antisense expression. To simulate such a situation, we removed the strand information for the DeLi-seq results, leading to 110 false-negatives (or 23% of all differentially expressed genes) and 27 false-positives (Supplementary information file, Figure S13). Notably, the false-negative genes are highly enriched in cellular functions involved in stress response (*p* < 9.2E-12), suggesting that they are true positives and would be missed by eliminating strand information. No functional enrichment can be detected for false positive genes, indicating that they are not involved in stress response. Together, these results demonstrate that strand-specific transcriptome sequencing can greatly improve the reliability of expression analyses, regardless of whether antisense transcripts are of interest or not.
