# Supplementary material for: The Prevalence and Regulation of Antisense Transcripts in Schizosaccharomyces pombe
Source: PLoS One. 2010 Dec 20;5(12):e15271. doi: 10.1371/journal.pone.0015271 (PMC3004915; doi:10.1371/journal.pone.0015271)
Supplement: Methods S1 — Supplementary Methods. (DOC) [file pone.0015271.s002.doc]

**Supplementary information file:**

**Supplementary Methods**

**Yeast strain and treat conditions.**

*S. pombe* stain Kfy1 was cultured in 100 ml YES medium at 32ºC. Cells were harvested at log phase (OD_600_ = 0.8). For heat shock treatment, *S. pombe* cells were grown at 40ºC for an additional 15 min with shaking before harvest. Two biological replicates were prepared for both the rich medium and heat shock conditions.

**RNA preparation**

Total RNA was purified from fission yeast cells using hot phenol protocol. To remove DNA contamination, RNeasy Mini kit (QIAGEN) was used according to the manufacturer’s protocol and an on-column DNase I digestion step was included.

**PolyA+ selection**

PolyA+ RNA was enriched using Dynabeads Oligo(dT)_25_ (Invitrogen). Briefly, 150 μg total RNA was resuspended in 400 μl binding buffer (20 mM Tris-HCl, pH 7.5, 1.0 M LiCl, 2 mM EDTA, 1% LiDS, 0.1% Trion X-100) and heated at 65°C for 2 min to disrupt the RNA secondary structures. After snap cool down, 200 μl Dynabeads was added followed by incubation at 50°C for 5 min. Incubation at a higher than suggested temperature helps remove the non-specific binding of rRNA. The resulting beads were then washed 3 times with Washing Buffer B (10 mM Tris-HCl, pH 7.5, 0.15 M LiCl, 1 mM EDTA, 0.1% LiDs (capital S??), 0.1% Triton X-100). The RNA fraction bound to the beads was then eluted with 10 mM Tris-HCl (RNase-free) by heating at 75-80°C for 2 min. The enrichment procedure was performed twice to obtain high-quality PolyA+ RNA.

**Second strand synthesis**

Second-strand synthesis was carried out in a 50 μl reaction, containing 1x 2nd-stand buffer (500 mM Tris-HCl, pH7.8, 50 mM MgCl2 and 10 mM DTT), 40.5 μl cDNA and 15 pmol dNTP. After incubation on ice for 5 min, 25 units of DNA polymerase I (NEB) and 1 unit of RNase H (Invitrogen) were added, followed by incubation at 15°C for 2.5 hours. The resulting double-stranded cDNAs were further polished by adding 3 units of T4 DNA polymerase (NEB) and incubation at 15°C for an additional 5 min. The end-repaired cDNA fragments were ethanol precipitated using GlycoBlue (Ambion) as the carrier.

**Directional ligation**

Double-stranded cDNA fragments were A-tailed and digested with I-Sce I (NEB) to produce asymmetrical termini. Directional ligation was subsequently performed in a 20 μl reaction by adding 3.75 nmol Solexa adaptors A and B (see below), 2 μl of 10x T4 DNA ligase buffer (NEB) and 2 μl of T4 DNA ligase (NEB; 2000 units/μl). After incubation at room temperature for 90 min, the ligation products were purified by ZYMO clean & concentrator-5 kit, followed by size-selection using 6% polyacrylamide gel to obtain 200-300 bp DNA fragments. The gel slice was incubated in 1x gel elution buffer (0.1% SDS, 0.3 M NaAc, pH5.2) for at least 4 hours, and the eluted DNA was recovered by ethanol precipitation. The sequences of Solexa adaptors are shown below:

Adaptor A:

5’- ACCGAGATCTACACTCTTTCCCTACACGACGCTCTTCCGATCT-3’

3’- ddCTGGCTCTAGATGTGAGAAAGGGATGTGCTGCGAGAAGGCTAGp-5’

Adaptor B:

5’- GATCGGAAGAGCTCGTATGCCGTCTTCTGCTTGddC-3’

3’-TATTCTAGCCTTCTCGAGCATACGGCAGAAGACGAAC -5’

**Low-cycle PCR**

DeLi-seq library was amplified by low-cycle suppression PCR before Illumina/Solexa sequencing. A 50μl reaction was assembled, which contains size-selected DNA, 1x HF buffer (Finnzymes), 1 nmol dNTP, 25 pmol of the forward primer (5’-AATGATACGGCGACCACCGAGA-3’) and the reverse primer (5’-CAAGCAGA AGACGGCATACGAG-3’), 2.5 pmol of the ‘add-on’ primer (5’-AATGATACGGCGACCACCGAGATCTA CACTCTTTCCCTACA-3’) and 0.5 μl of Phusion Hot Start High-Fidelity DNA Polymerase (Finnzymes). Thermal cycling was carried out as the following: 98°C for 30s; 2 cycles of 98°C for 10s, 66°C for 30s and 72°C for 30s; 12 cycles of 98°C for 10s, 68°C for 30s and 72°C for 30s; 72°C for 10 min; hold at 10°C. The PCR products (or final DeLi-seq library) were purified by ZYMO clean & concentrator-5 kit and quantified by Qubit Fluorometer (Invitrogen) before Illumina/Solexa sequencing.

**Identification of novel introns**

Novel introns were identified based on uniquely mapped reads that contain a gap when mapping to the *S. pombe* genome with BLAT algorithm and the gaps do not fall within the known intron. Three criteria were used to define a novel intron: (a) the boundary of the gap must follow GT-AG rule; (b) the gap must be less than 2,000 bp; (c) there are at least two non-redundant junction reads that support the putative novel intron.
