# Supplementary figures and images for: The Prevalence and Regulation of Antisense Transcripts in Schizosaccharomyces pombe
Source: PLoS One. 2010 Dec 20;5(12):e15271. doi: 10.1371/journal.pone.0015271 (PMC3004915; doi:10.1371/journal.pone.0015271)

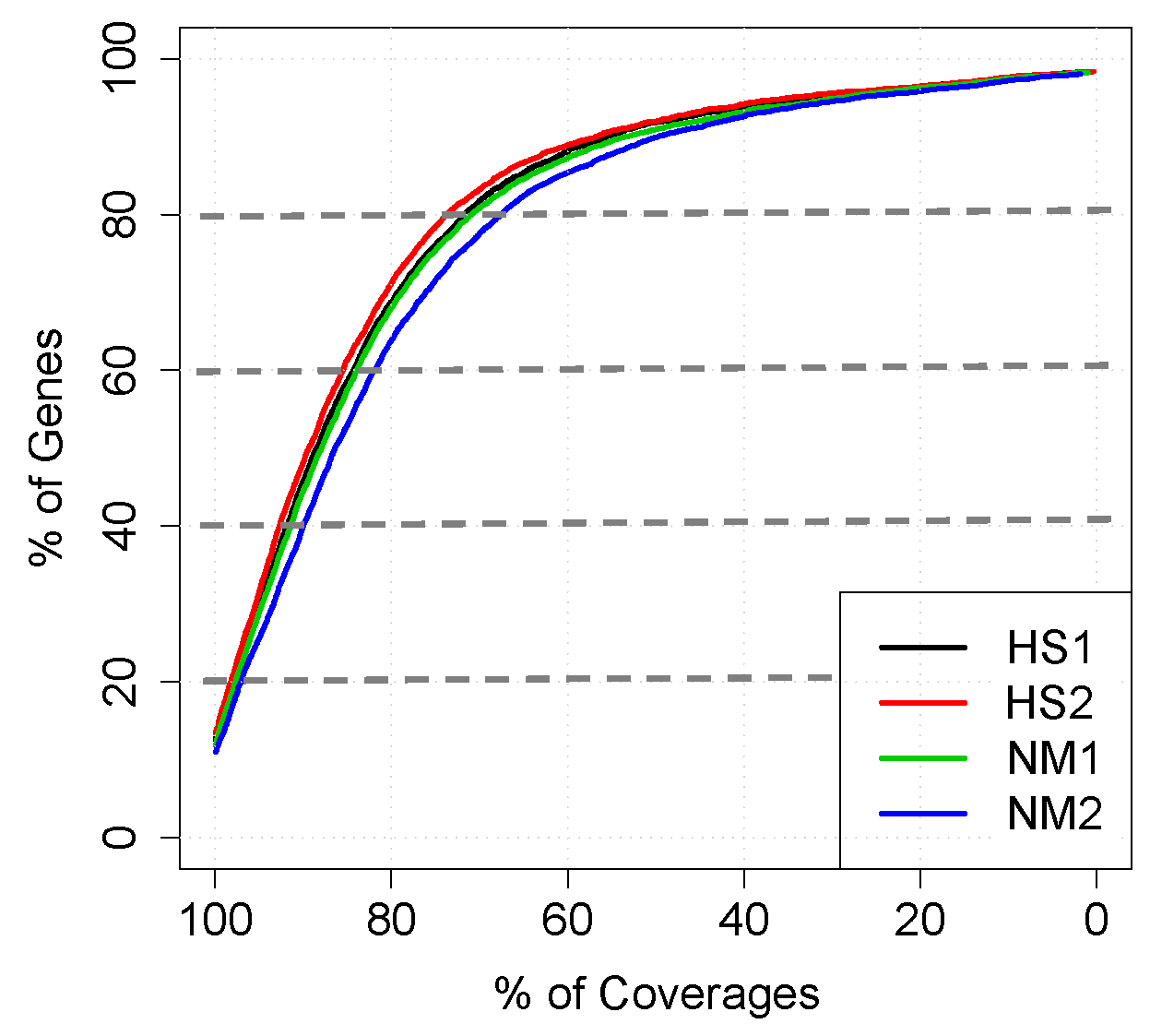

Supplement: Figure S1 — Overall coverage of sense transcripts. For each annotated protein-coding gene in S. pombe, the coverage at the nucleotide level was computed based on the DeLi-seq reads uniquely mapped to the locus in the sense orientation. Cumulative plot is used to show the percentage of genes that passes each respective coverage threshold. Four DeLi-seq libraries were analyzed separately and the results showed that they have comparable coverage depth. (TIF) [file pone.0015271.s003.tif]

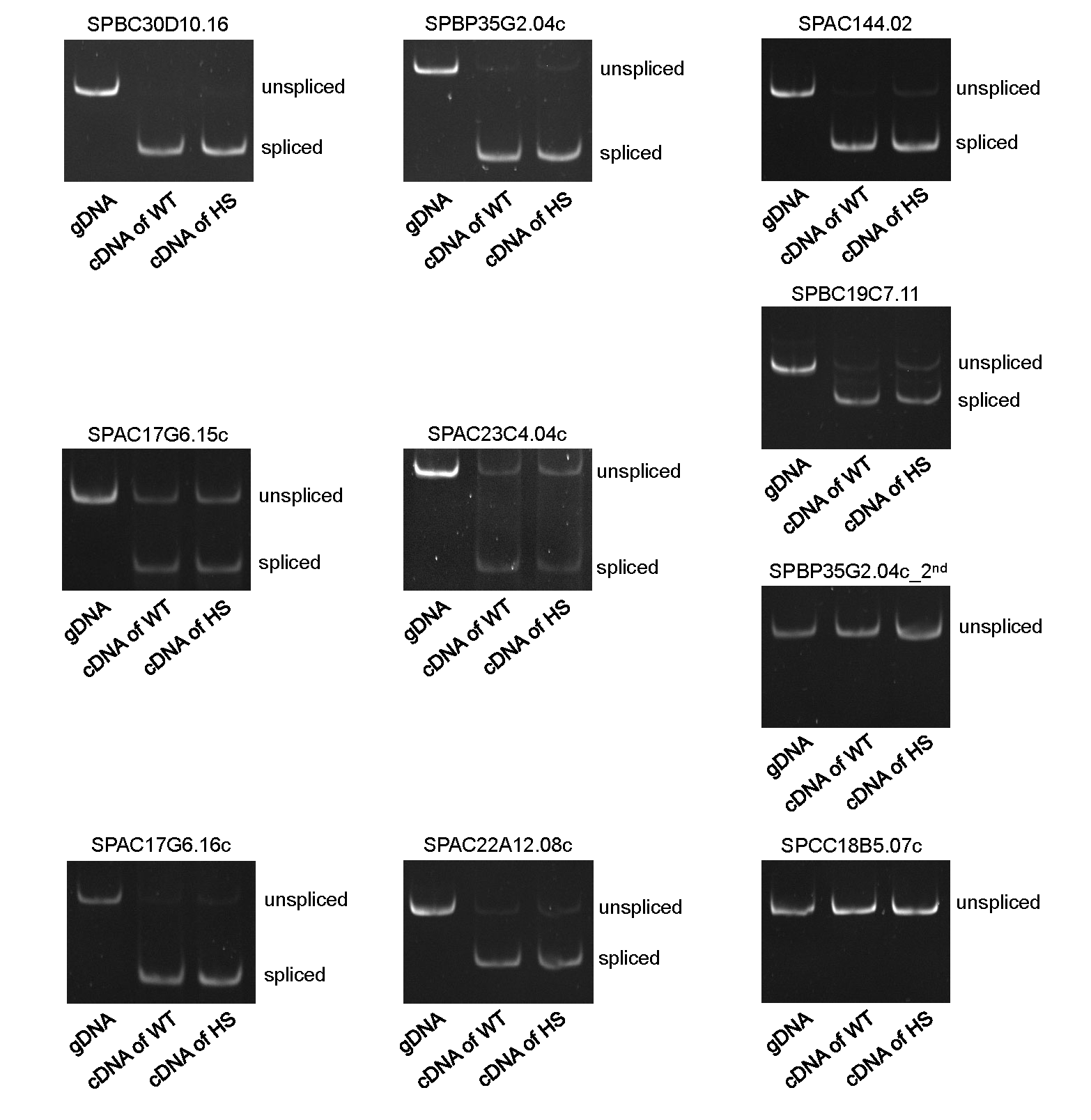

Supplement: Figure S2 — Validation of novel introns. 10 candidate novel introns were selected for validation. For each candidate, a pair of gene-specific primers was designed upstream and downstream of the putative intron. RT-PCR was performed with the RNA samples obtained from normal or heat shock condition. Genomic DNA was used as a negative control, which gives rise to unspliced products. For 8 out the 10 cases, spliced products with an expected size were observed. In the case of SPBP35G2.04c, two novel introns were identified by DeLi-seq, and one of them was randomly selected for validation. (TIF) [file pone.0015271.s004.tif]

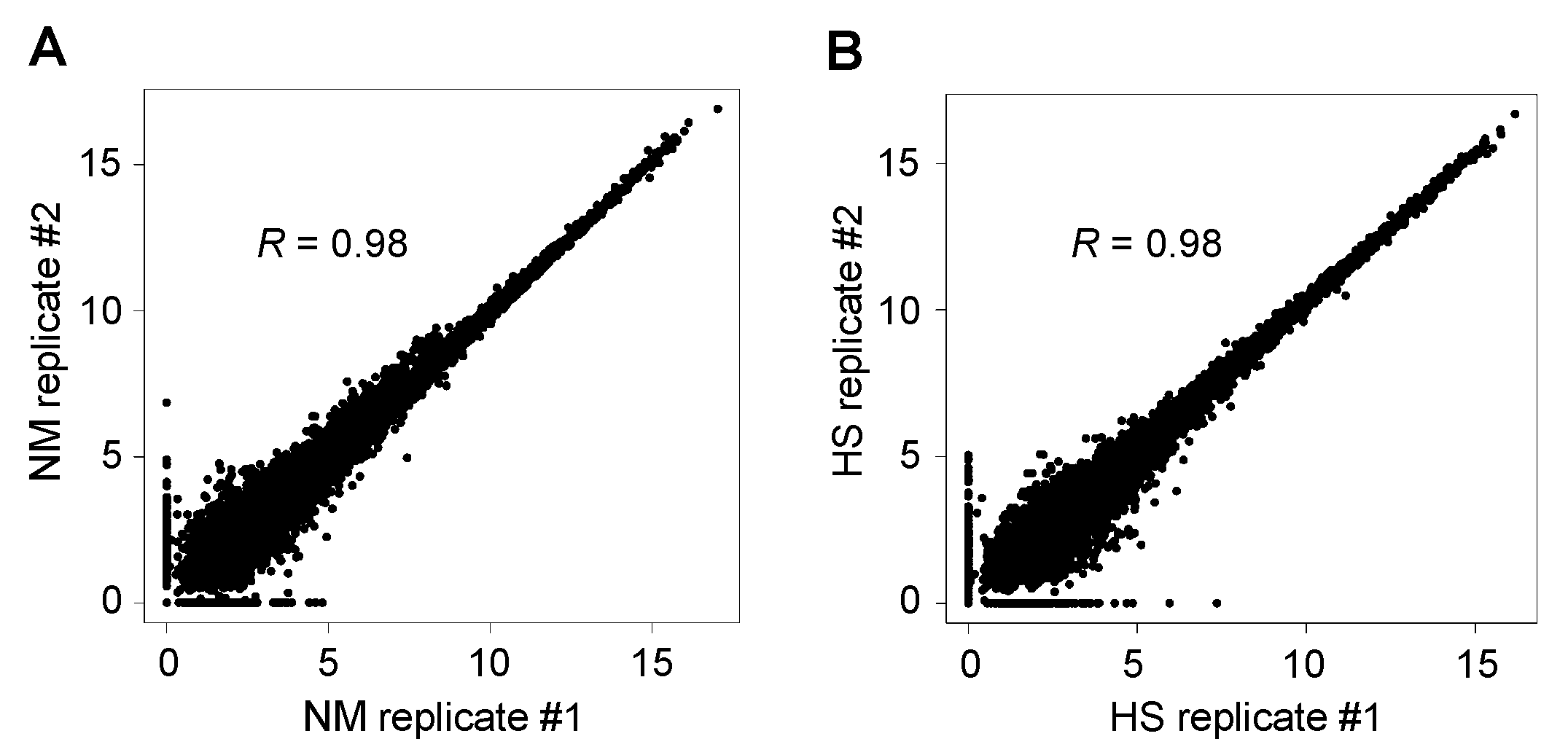

Supplement: Figure S3 — Reproducibility of the DeLi-seq method. The count of uniquely mapped reads for each annotated locus was determined and normalized to the total number of reads of each library. Sense and antisense transcripts of each locus were treated as separated data points. Correlation coefficient was then computed between the two biological replicates of either normal (A) or heat shock (B) condition. (TIF) [file pone.0015271.s005.tif]

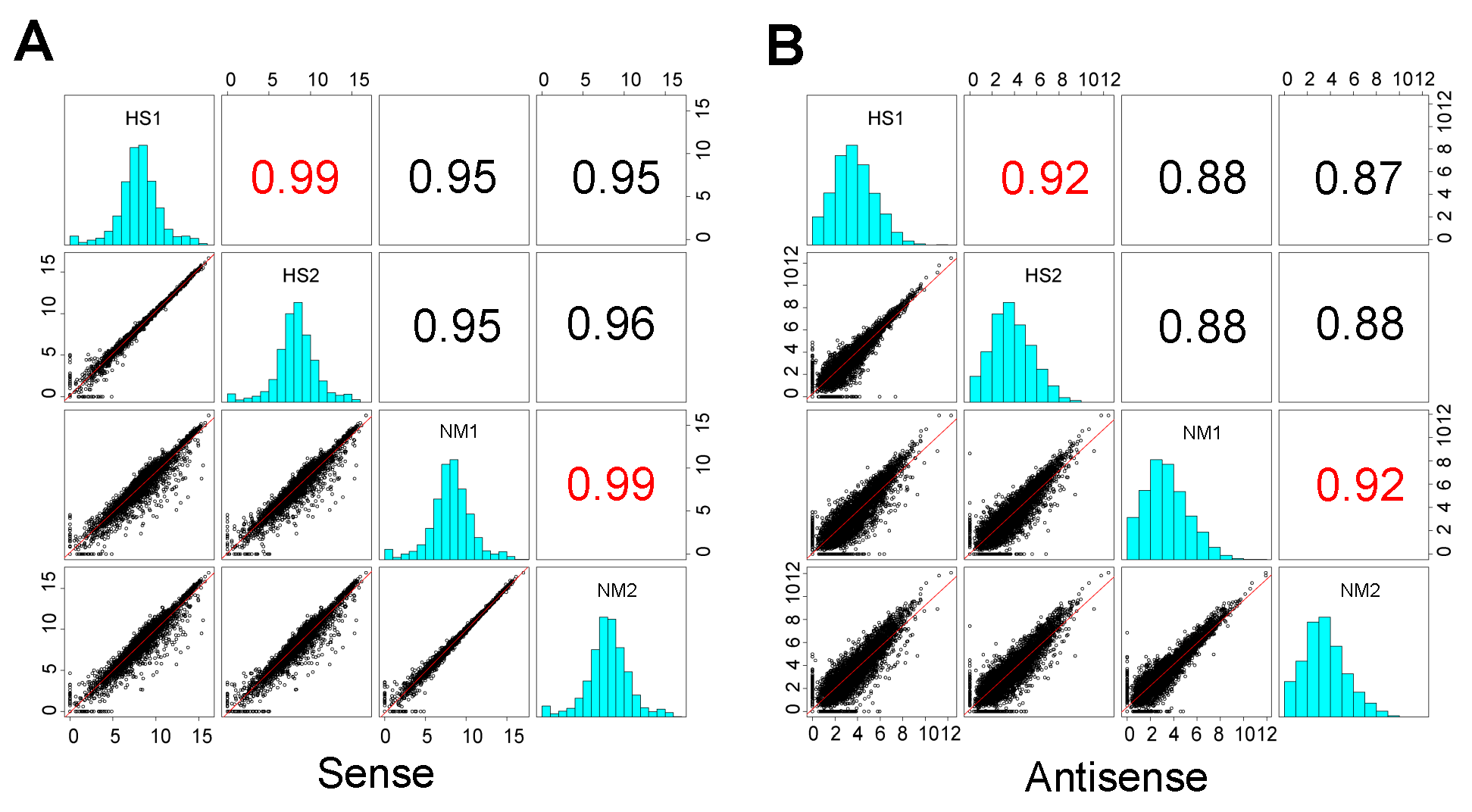

Supplement: Figure S4 — Reproducibility of sense or antisense read counts. Pairwise comparison of the normalized expression level of sense (A) or antisense (B) transcripts among four DeLi-seq libraries. The histograms on the main diagonal represent the expression distribution of individual libraries. Each scatter plot was generated based on the normalized gene expression levels obtained from the two corresponding libraries. Pearson correlation coefficients are shown for all possible library pairs. (TIF) [file pone.0015271.s006.tif]

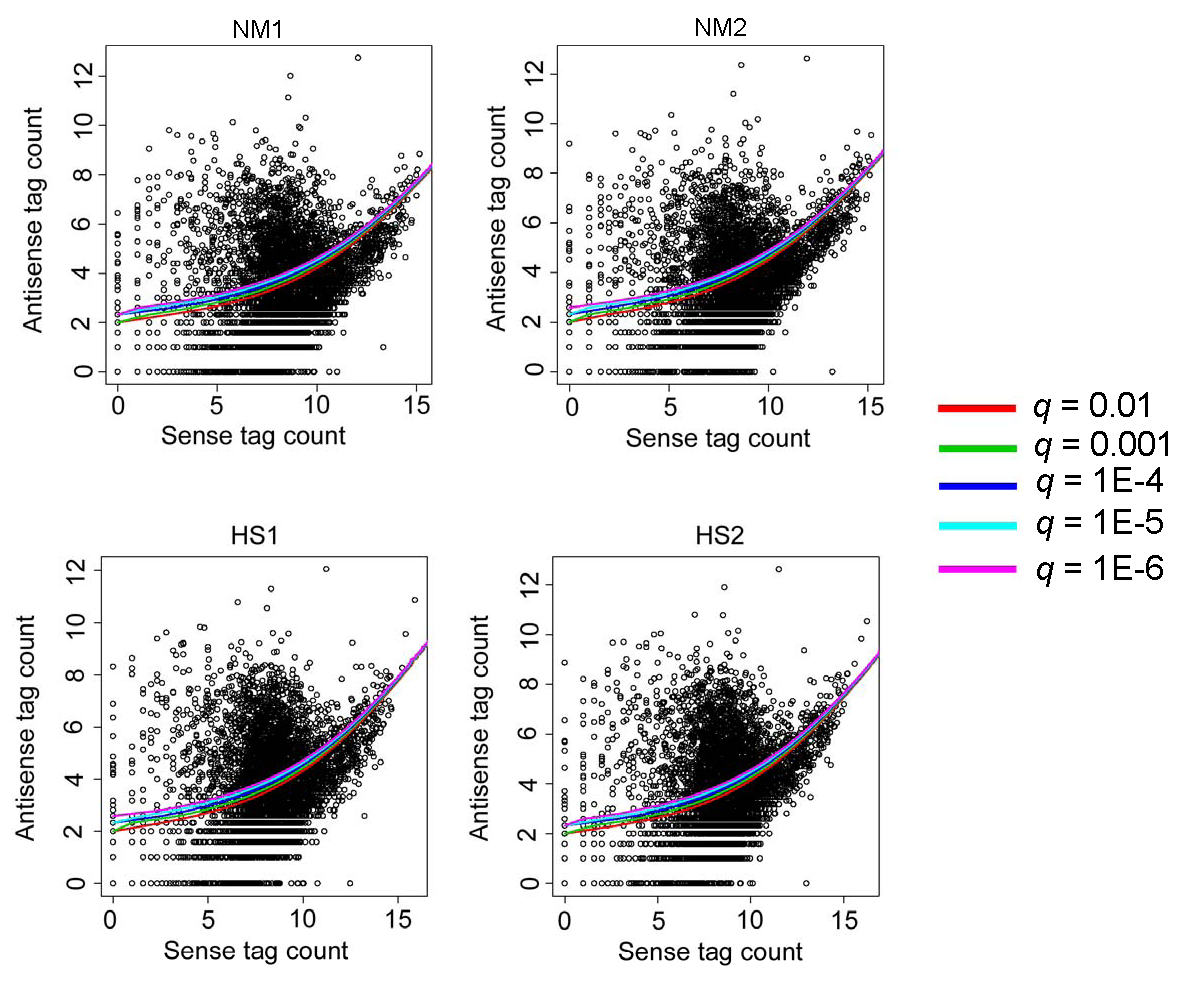

Supplement: Figure S5 — Identification of gene loci with high-confidence antisense expression. Since the overall log2(S/AS) follows a negative binomial distribution (Figure 2A), we thus used negative binomial statistics to remove low-confidence call of antisense transcripts. The background rate of each library was experimentally defined based on the splicing junction reads. Five different thresholds were used as indicated. (TIF) [file pone.0015271.s007.tif]

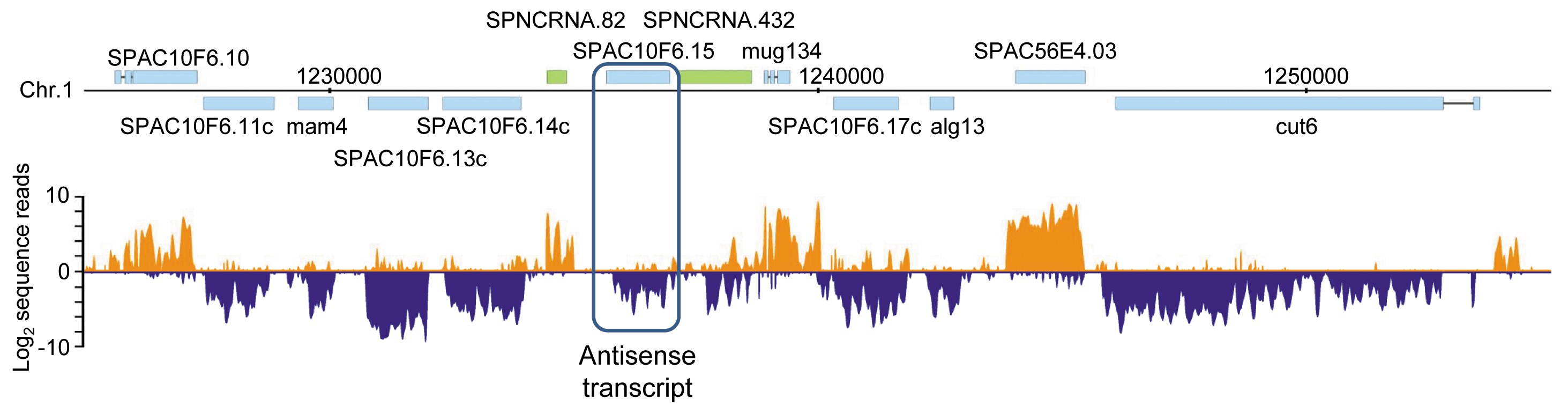

Supplement: Figure S6 — Visualization of DeLi-seq results. A 30 kb genomic region is shown with known annotations (upper panel), including protein-coding genes (light blue) and ncRNAs (green). In the lower panel, read counts from the top strand (orange) and bottom strand (dark blue) are shown separately. For SPAC10F6.15 locus, the level of antisense transcripts is much higher than that of sense transcripts (open box). (TIF) [file pone.0015271.s008.tif]

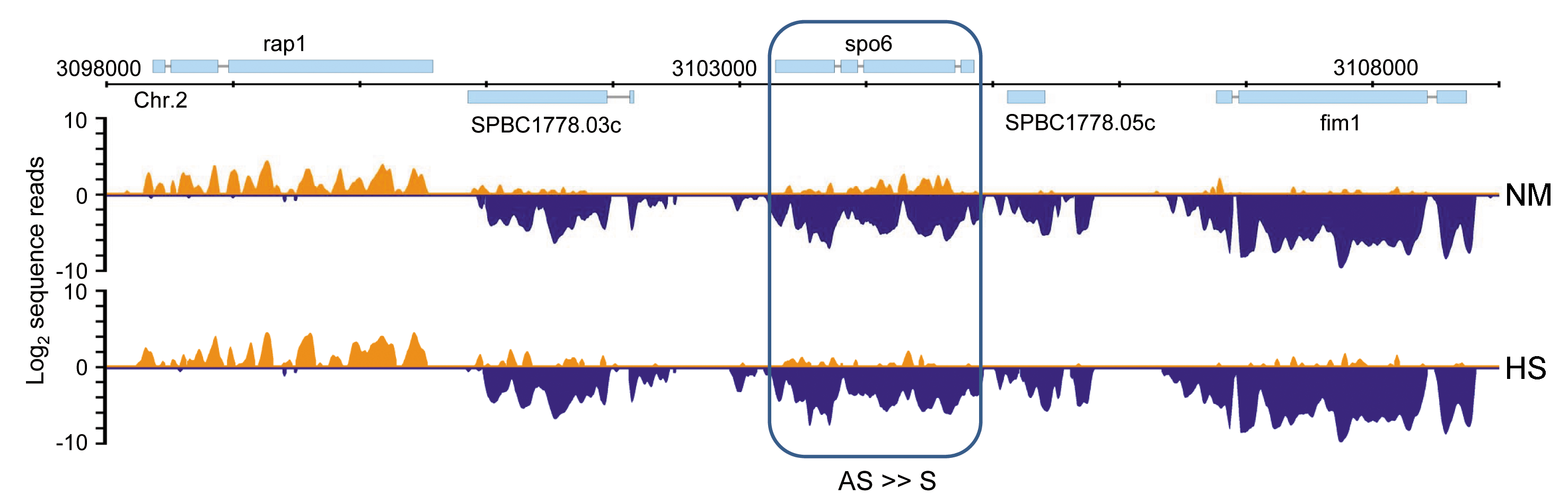

Supplement: Figure S7 — Dominant antisense expression at the spo6 locus. Spo6 gene is encoded on the Watson strand based on genome annotation. However, majority of the reads were mapped to the Crick strand in this locus under both normal and heat shock conditions. (TIF) [file pone.0015271.s009.tif]

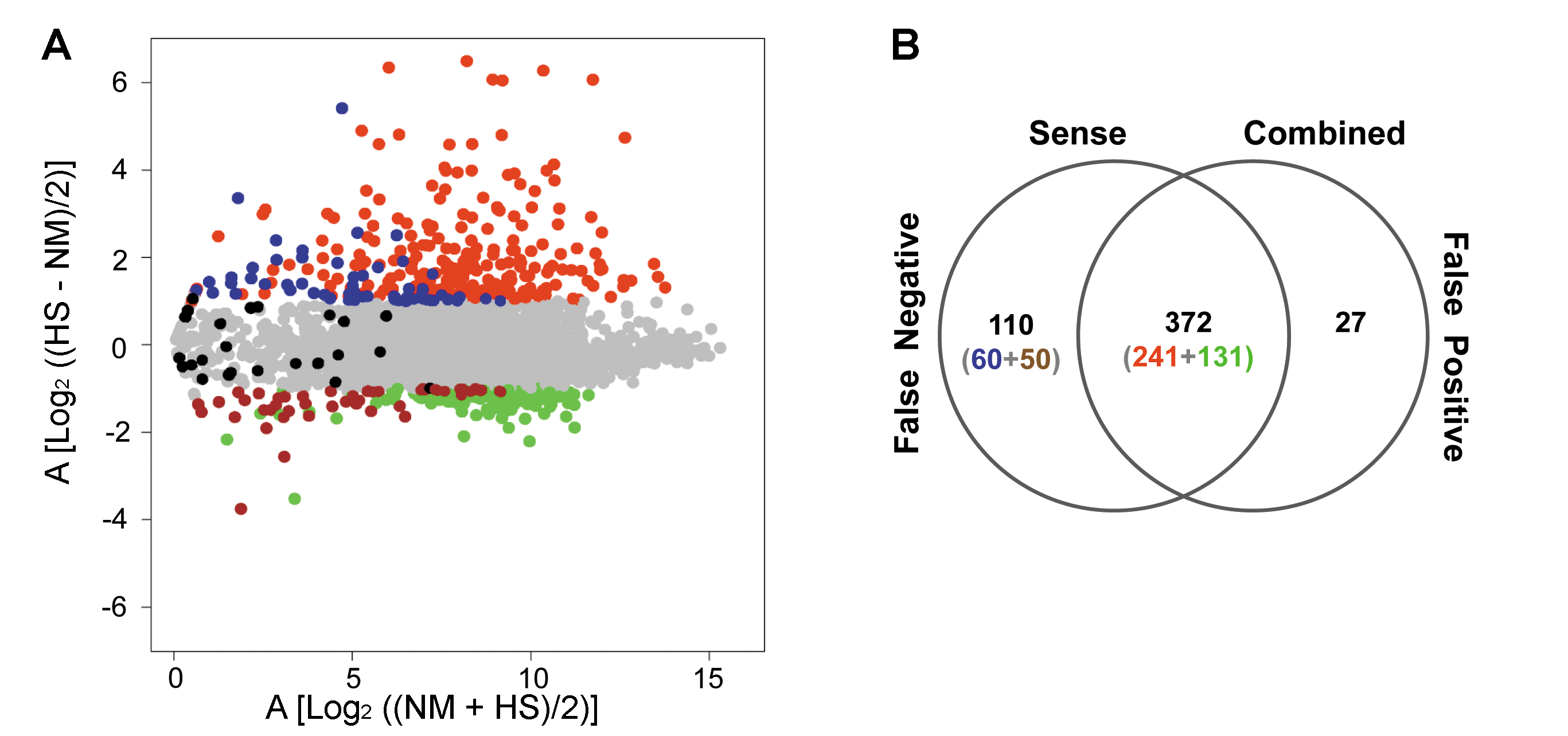

Supplement: Figure S8 — Comparison between DeLi-seq and HybMap results. For DeLi-seq method, the relative expression level of each transcript was computed based on normalized read count. HybMap data was downloaded from http://bioserver.hci.utah.edu/SupplementalPaperInfo/2008/Dutrow_NatGen_PombeTranscriptome/. The data set contains the expression values that were computed based on the probe intensity subtracted against the background (intergenic regions). Pearson correlation coefficient (R) was computed for both sense (A) and antisense (B) transcripts. (TIF) [file pone.0015271.s010.tif]

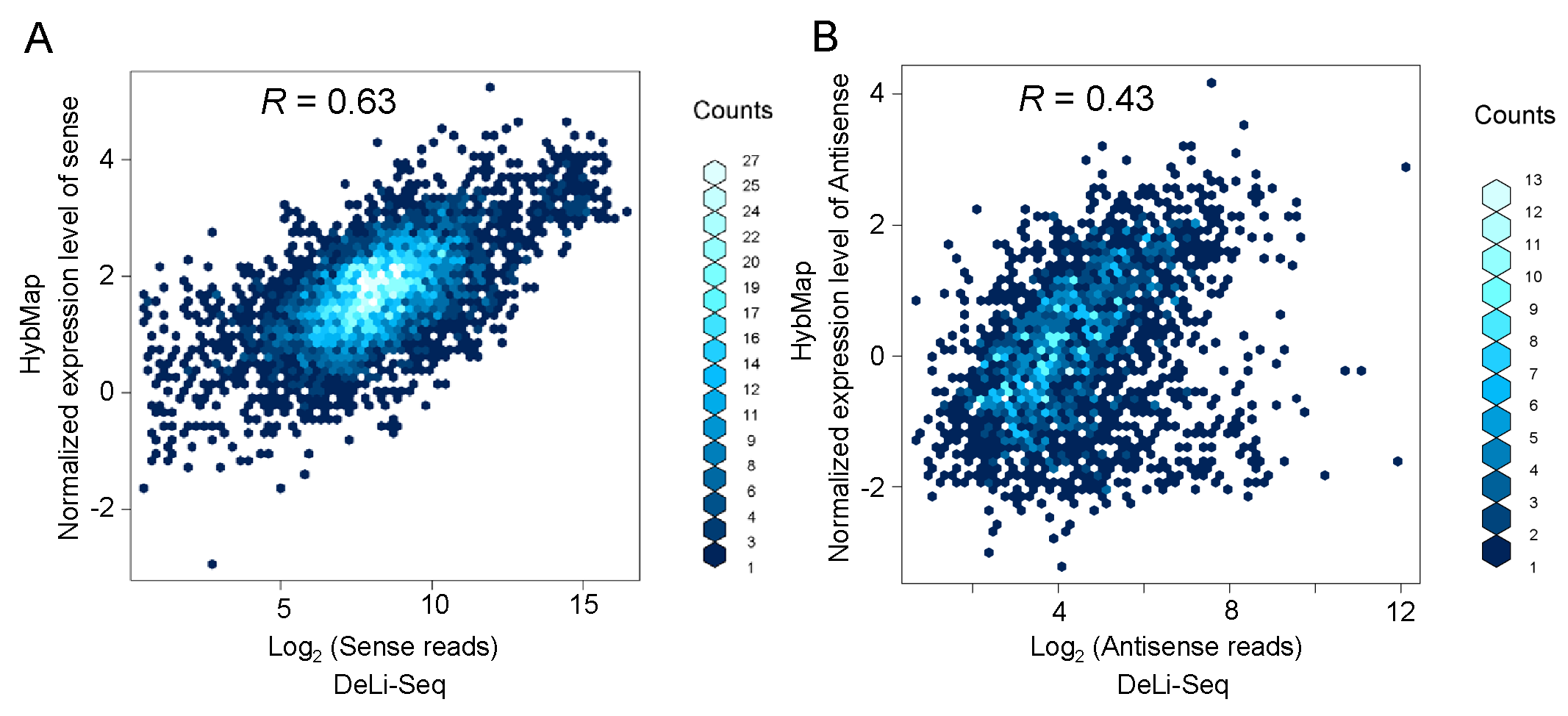

Supplement: Figure S9 — Validation of antisense expression by strand-specific RT-PCR. 16 genes were randomly selected to examine antisense expression. These genes are broadly divided into four different categories based on the ratio of sense (S) and antisense (AS) transcripts (PP: S≥ AS in both NM and HS; PN: S≥ AS in NM and S< AS in HS; NP: S< AS in NM and S≥ AS in HS; NN: S< AS in both NM and HS). To carry out strand-specific RT-PCR, either the forward or reverse primer was added at the RT step, which specifically amplifies the antisense or sense transcripts, respectively. As a positive control, both primers were added at the RT step (T). In addition, RT reaction was also performed without any primer to serve as a negative control (−). The final PCR products were resolved by agarose gel electrophoresis, and each of the primer pairs gave rise to a specific band with expected size. (TIF) [file pone.0015271.s011.tif]

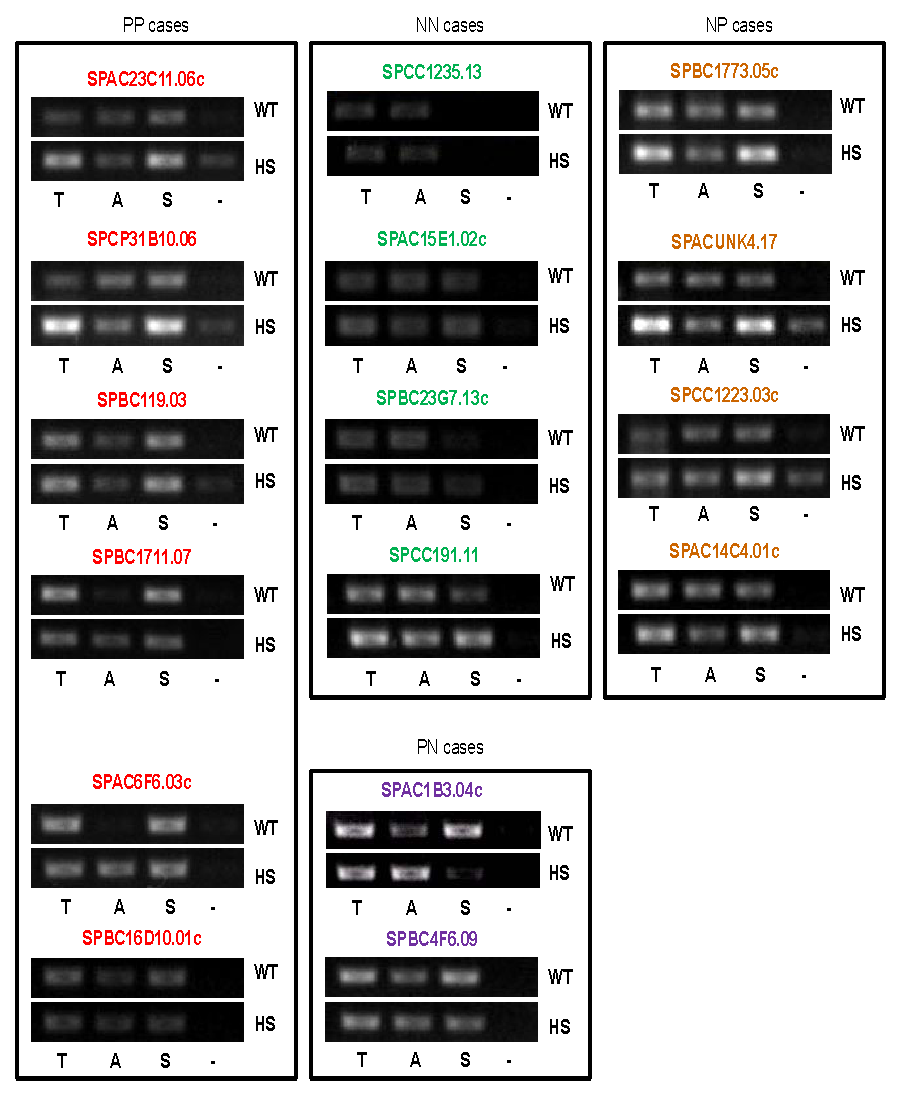

Supplement: Figure S10 — Comparison of antisense expression level in differentially expressed genes. Differentially expressed genes were divided into two groups based on sense-antisense expression correlation. The correlated group (Cor) contains sense-antisense pairs with correlated or anti-correlated expression patterns. The non-correlated group (Non-Cor) group consists gene loci for which sense and antisense transcripts are independently regulated. The absolute antisense expression level (A) or relative antisense/sense ratio (B) was then compared between the two groups by one-way ANOVA test. No significant difference can be detected between the two subcategories in terms of antisense expression level (p = 0.125) or antisense/sense ratio (p = 0.776). (TIF) [file pone.0015271.s012.tif]

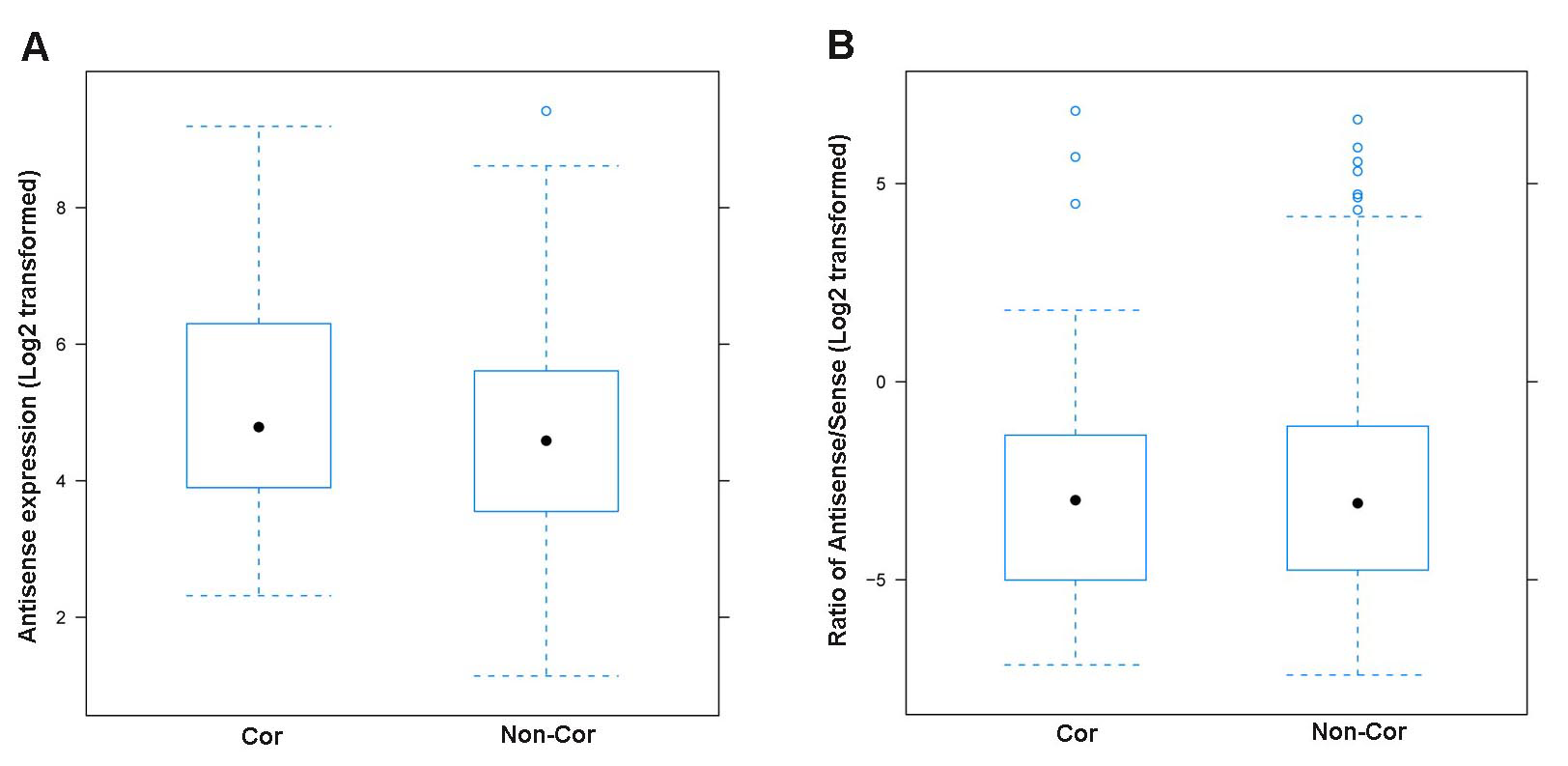

Supplement: Figure S11 — Bidirectional promoter leads to antisense transcription in tandem gene pair. (A) A Schematic diagram of the Wis2-Guf1 locus. The sense transcripts of these two genes (solid boxes) are encoded on the negative strand of chromosome 1, and organized in a tandem orientation. Based on the DeLi-seq results, the expression of Guf1 antisense transcripts (dashed box) are increased under the heat shock condition and the increase is coordinated with Wis2 sense transcript. Wis2 encodes a peptidyl-prolyl isomerase required for protein unfolding, transport and assembly. The Guf1 sense transcript encodes a mitochondrial matrix GTPase associated with mitochondrial ribosome, and is known to be down-regulated by heat shock. (B) Validation of DeLi-seq results for the Wis2-Guf1 locus by strand-specific RT-PCR. T, combined level of sense and antisense transcripts; A: antisense transcripts; S: sense transcripts; -, negative control, for which no primer was added during reverse transcription. (C) A conventional heat shock element (HSE) with multiple GAA blocks is identified in the candidate bidirectional promoter region. Both sense transcript of Wis2 and antisense transcript of Guf1 have their own TATA-box (black block). (TIF) [file pone.0015271.s013.tif]

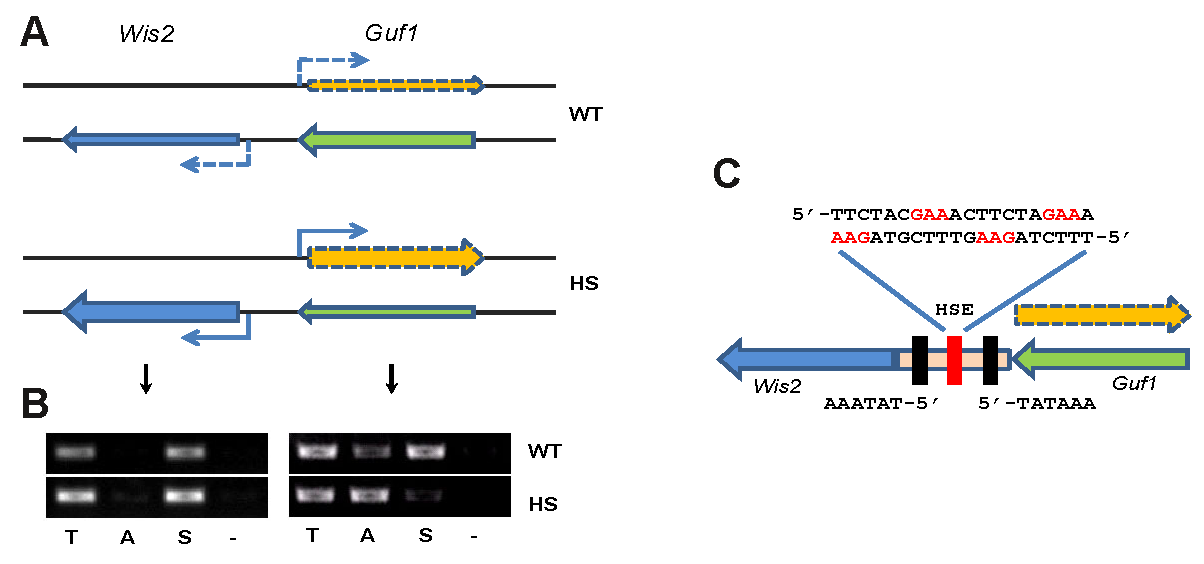

Supplement: Figure S12 — Antisense transcripts derived from transcriptional read-though at a convergent locus. (A) Schematic diagram of the SPBC365.11 and SPBC365.12c loci. The two sense transcripts (solid box) are coded on different strands and organized in a convergent orientation (tail-to-tail). The expression level of both SPBC365.12c and the antisense transcripts to SPBC265.11 (dashed box) are induced by heat shock. In contrast, the sense transcript of SPBC365.11 is transiently down-regulated under the heat shock condition. (B) Heat shock induced transcriptional readthrough is confirmed by strand-specific RT-PCR. T, Total level of sense and antisense transcripts; AS, expression level of antisense transcripts; S, expression level of sense transcripts; -, negative control, which contained no primer during reverse transcription. In order to detect readthrough transcripts, a primer pair was used which spans the two annotated genes; the relative locations of these two primers are shown. At the RT step, only left (L) or right (R) primer was added to detect readthrough transcript of right (SPBC365.12c) or left (SPBC365.11) gene. The readthrough transcript of SPBC365.12c gene was apparent under the heat shock condition. (TIF) [file pone.0015271.s014.tif]

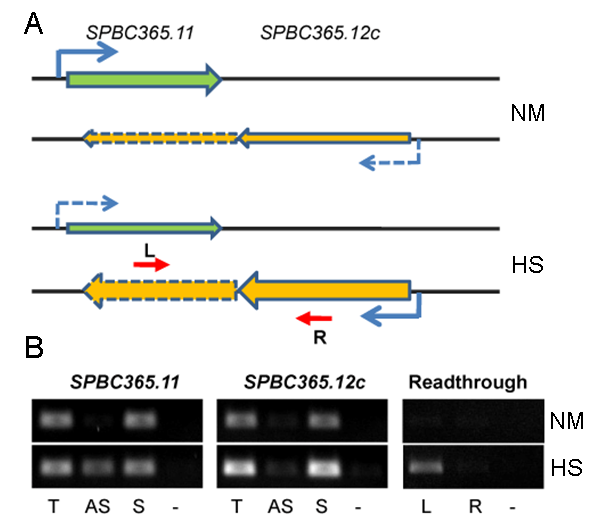

Supplement: Figure S13 — Differentially expressed sense and antisense transcripts identified by DeLi-seq. (A) Differentially expressed sense transcripts (q<0.05 and >2-fold change) are shown as colored dots in a MA plot. Up-regulated (red and blue) and down-regulated (green and magenta) are shown. False negative (blue and magenta) and false positive (black) genes were determined assuming strand information is not provided. (B) The number of false positives and false negatives obtained in (A) are color coded and shown in a Venn diagram. (TIF) [file pone.0015271.s015.tif]
