# Supplementary material for: The Prevalence and Regulation of Antisense Transcripts in Schizosaccharomyces pombe
Source: PLoS One. 2010 Dec 20;5(12):e15271. doi: 10.1371/journal.pone.0015271 (PMC3004915; doi:10.1371/journal.pone.0015271)
Supplement: Table S1 — Mapping efficiency of sequencing reads. (DOC) [file pone.0015271.s016.doc]

**Supplementary information file:**

**Table S1.** Mapping efficiency of sequencing reads

| **Sample ID** | **Total Reads** | **Mapped reads** | **% mapped** | **Uniquely mapped reads** | **% uniquely mapped** | **Reads mapped to multiple Locations** | **Non-redundant reads** |
| --- | --- | --- | --- | --- | --- | --- | --- |
| NM1 | 8,491,844 | 8,271,679 | 97.4% | 6,180,018 | 72.8% | 2,091,661 | 2,266,500 |
| NM2 | 8,110,689 | 7,816,344 | 96.4% | 5,829,520 | 71.9% | 1,986,824 | 1,681,750 |
| HS1 | 7,215,858 | 7,026,772 | 97.4% | 5,616,231 | 77.8% | 1,410,541 | 2,041,662 |
| HS2 | 8,494,538 | 8,209,488 | 96.6% | 6,547,598 | 77.1% | 1,661,890 | 1,895,955 |
