# Supplementary material for: The Prevalence and Regulation of Antisense Transcripts in Schizosaccharomyces pombe
Source: PLoS One. 2010 Dec 20;5(12):e15271. doi: 10.1371/journal.pone.0015271 (PMC3004915; doi:10.1371/journal.pone.0015271)
Supplement: Table S2 — The reads mapped to the exon-exon junctions in sense and antisense transcripts. (DOC) [file pone.0015271.s017.doc]

**Supplementary information file:**

**Table S2. The reads mapped to the exon-exon junctions in sense and antisense transcripts**

| **Sample ID** | **Sense junction hits GT-AG** | **Antisense junction hits CT-AC** | **GT-AG/TC-AC** | **False positive rate  of antisense** |
| --- | --- | --- | --- | --- |
| NM1 | 79,108 | 322 | 245.68 | 0.41% |
| NM2 | 68,169 | 412 | 165.46 | 0.60% |
| HS1 | 59,038 | 280 | 210.85 | 0.47% |
| HS2 | 64,948 | 242 | 268.38 | 0.37% |
