# Supplementary material for: The Prevalence and Regulation of Antisense Transcripts in Schizosaccharomyces pombe
Source: PLoS One. 2010 Dec 20;5(12):e15271. doi: 10.1371/journal.pone.0015271 (PMC3004915; doi:10.1371/journal.pone.0015271)
Supplement: Table S3 — Uniquely mapped reads in protein-coding region and ncRNAs. (DOC) [file pone.0015271.s018.doc]

**Supplementary information file:**

**Table S3. Uniquely mapped reads in** protein-coding region and ncRNAs

| **Sample ID** | **Protein-coding genes*** | | | | | | | | **ncRNAs** | | **Total Antisense** | |
| --- | --- | --- | --- | --- | --- | --- | --- | --- | --- | --- | --- | --- |
|  | **5’ UTR** | | **CDS** | | **Intron** | | **3’ UTR** | |  |  |  |  |
|  | **S** | **AS** | **S** | **AS** | **S** | **AS** | **S** | **AS** | **S** | **AS** | **Reads** | **%** |
| NM1 | 66,668 | 1,267 | 5,361,064 | 154,080 | 20,445 | 5,191 | 75,338 | 2,392 | 14,805 | 6,833 | 169,763 | 2.97% |
| NM2 | 58,506 | 1,174 | 5,382,515 | 166,055 | 20,700 | 5,649 | 70,759 | 2,839 | 15,017 | 6,396 | 182,113 | 3.18% |
| HS1 | 62,440 | 1,147 | 4,794,200 | 169,149 | 19,897 | 5,349 | 69,648 | 2,691 | 25,397 | 6,337 | 184,673 | 3.58% |
| HS2 | 72,601 | 1,323 | 5,983,806 | 192,568 | 24,018 | 6,261 | 76,996 | 2,834 | 26,358 | 6,618 | 209,604 | 3.28% |

*The uniquely mapped reads fall in protein-coding gene regions are divided into four categories: 1) 5' untranslated regions (5’ UTRs); 2) coding regions (CDS); 3) introns; and 4) 3’ UTRs. For each category, the corresponding reads were further divided into the sense (S) and antisense (AS) orientations.
