# Supplementary material for: The Prevalence and Regulation of Antisense Transcripts in Schizosaccharomyces pombe
Source: PLoS One. 2010 Dec 20;5(12):e15271. doi: 10.1371/journal.pone.0015271 (PMC3004915; doi:10.1371/journal.pone.0015271)
Supplement: Table S4 — GO analysis of genes with AS ≥S in both NM and HS. (DOC) [file pone.0015271.s019.doc]

**Supplementary information file:**

**Table S4. GO analysis of genes with AS ≥ S in both NM and HS**

| **GO ID** | **Term** | **Annotated** | **Significant** | **Expected** | ***p* value** |
| --- | --- | --- | --- | --- | --- |
| GO:0007126 | meiosis | 175 | 35 | 7.98 | 5.10E-11 |
| GO:0045132 | meiotic chromosome segregation | 57 | 13 | 2.6 | 1.10E-06 |
| GO:0032005 | signal transduction involved in conjugation with cellular fusion | 18 | 5 | 0.82 | 0.001 |
| GO:0030437 | ascospore formation | 95 | 12 | 4.33 | 0.0011 |
| GO:0015718 | monocarboxylic acid transport | 2 | 2 | 0.09 | 0.0021 |
| GO:0007131 | meiotic recombination | 57 | 8 | 2.6 | 0.0039 |
| GO:0034065 | replication fork processing at rDNA locus | 3 | 2 | 0.14 | 0.006 |
| GO:0006284 | base-excision repair | 9 | 3 | 0.41 | 0.0064 |
| GO:0006310 | DNA recombination | 98 | 14 | 4.47 | 0.0088 |
