# Supplementary material for: The Prevalence and Regulation of Antisense Transcripts in Schizosaccharomyces pombe
Source: PLoS One. 2010 Dec 20;5(12):e15271. doi: 10.1371/journal.pone.0015271 (PMC3004915; doi:10.1371/journal.pone.0015271)
Supplement: Table S5 — GO analysis of genes with AS ≥S in NM but not HS condition. (DOC) [file pone.0015271.s020.doc]

**Supplementary information file:**

**Table S5. GO analysis of genes with AS ≥ S in NM but not HS condition**

| **GO.ID** | **Term** | **Annotated** | **Significant** | **Expected** | ***p* value** |
| --- | --- | --- | --- | --- | --- |
| GO:0033554 | cellular response to stress | 446 | 26 | 4.97 | 1.20E-13 |
| GO:0006865 | amino acid transport | 36 | 3 | 0.4 | 0.0072 |
| GO:0055085 | transmembrane transport | 264 | 8 | 2.94 | 0.0083 |
