# Supplementary material for: The Prevalence and Regulation of Antisense Transcripts in Schizosaccharomyces pombe
Source: PLoS One. 2010 Dec 20;5(12):e15271. doi: 10.1371/journal.pone.0015271 (PMC3004915; doi:10.1371/journal.pone.0015271)
Supplement: Table S6 — Primers used for strand-specific RT-PCR and qPCR. (DOC) [file pone.0015271.s021.doc]

**Supplementary information file:**

**Table S6.** Primers used for strand-specific RT-PCR and qPCR

|  | **Systematic gene name in *S. pombe*** | **Primers** | **Sequence of primers (5’ to 3’)** |
| --- | --- | --- | --- |
| PP | SPAC23C11.06c | Forward | ATGTCAGCGGTCCGTATGTG |
|  |  | Reverse | GGTTGATCCCCAAAGGTGAA |
|  | SPCP31B10.06 | Forward | AAGTCCGGCTCAGAGCGTAG |
|  |  | Reverse | ACGGTGAAGCACAACATTGC |
|  | SPBC119.03 | Forward | GTGAACGGAAGCCGAAAATC |
|  |  | Reverse | CCGGCCAACTTAACCAACTC |
|  | SPBC1711.07 | Forward | CAAGACCCAGCACGATGAAA |
|  |  | Reverse | CCTTACGCGAAACCATCACA |
|  | SPAC6F6.03c | Forward | CTGCCGCCTGGGTTAAAATA |
|  |  | Reverse | CCAGGAATGGGAGCAACATT |
|  | SPBC16D10.01c | Forward | TGCGGATAAAAGTCCCAAGG |
|  |  | Reverse | TGGGCAAGCCAAAGGTATTT |
| PN | SPAC1B3.04c | Forward | TCGAAACTGGGCTGTGATTG |
|  |  | Reverse | CTTCGGCACGAAAGTCAACA |
|  | SPBC4F6.09 | Forward | TGATGCCCAAGCGTTTAGAA |
|  |  | Reverse | GGGATTAGCGACGAACCAAA |
| NP | SPACUNK4.17 | Forward | TGCTCTCTTGTCCCCTCACA |
|  |  | Reverse | ATCAGGGAATCGCTTGAGGA |
|  | SPBC1773.05c | Forward | GCCCTTTACTCCCATTGCTG |
|  |  | Reverse | CCATTTCCAGCTTGCACAAA |
|  | SPCC1223.03c | Forward | AAATGGCTCATGGTGGTGTG |
|  |  | Reverse | AGAACCAGCAACCCAGTCGT |
|  | SPAC14C4.01c | Forward | TCAGAATCTGCCGTCGAAGA |
|  |  | Reverse | AAAGGCAGACCCATTTGCAC |
| NN | SPCC191.11 | Forward | CGAAGTTCATTGGGGTCACA |
|  |  | Reverse | GACGCTCAGCAACACCAGTC |
|  | SPAC15E1.02c | Forward | CACTGCACCTCACGTCGAAT |
|  |  | Reverse | AATGCAGCGATCGAGTACGA |
|  | SPBC23G7.13c | Forward | TGAACTCAAGCGCAAAGCTC |
|  |  | Reverse | ACAGTGTTCATGCCCGTCAC |
|  | SPCC1235.13 | Forward | TGACGTTGAGGCAGAATTGG |
|  |  | Reverse | AATCAAAGCTGCGAGGAAGG |
